# Methodological Developments for Metabolic NMR Spectroscopy from Cultured Cells to Tissue Extracts: Achievements, Progress and Pitfalls

**DOI:** 10.3390/molecules27134214

**Published:** 2022-06-30

**Authors:** Norbert W. Lutz, Monique Bernard

**Affiliations:** CRMBM, CNRS, Aix-Marseille University, 13005 Marseille, France; monique.bernard@univ-amu.fr

**Keywords:** in vitro NMR spectroscopy, tissue, cells, metabolism, extracts, heteronuclei

## Abstract

This is a broad overview and critical review of a particular group of closely related ex vivo and in vitro metabolic NMR spectroscopic methods. The scope of interest comprises studies of cultured cells and excised tissue, either intact or after physicochemical extraction of metabolites. Our detailed discussion includes pitfalls that have led to erroneous statements in the published literature, some of which may cause serious problems in metabolic and biological interpretation of results. To cover a wide range of work from relevant research areas, we consider not only the most recent achievements in the field, but also techniques that proved to be valid and successful in the past, although they may not have generated a very significant number of papers more recently. Thus, this comparative review also aims at providing background information useful for judiciously choosing between the metabolic ex vivo/in vitro NMR methods presented. Finally, the methods of interest are discussed in the context of, and in relation to, other metabolic analysis protocols such as HR-MAS and cell perfusion NMR, as well as the mass spectrometry approach.

## 1. Introduction

NMR analysis of cellular metabolism by studying physicochemical extracts of cultured cells and biological tissues is one of the oldest, best-established techniques in NMR metabolomics (sometimes referred to as metabonomics) [1]. Aqueous extracts, i.e., extracts of water-soluble compounds, result in aqueous metabolite solutions. Compared with metabolic NMR spectroscopy of intact tissue (in vivo or ex vivo), solution NMR spectroscopy has several benefits. The most important advantage is a greatly increased number of detectable and quantifiable metabolites due to (i) dramatically improved distinguishability of metabolite signals thanks to a spectacular increase in spectral resolution [2], and (ii) significantly augmented sensitivity based on intense signals from concentrated samples. Organic extracts, i.e., extracts of compounds soluble in organic solvents, result in solutions of metabolites that are not soluble in water, i.e., primarily lipids. The latter compounds only yield very broad NMR resonances when measured in intact tissue, rendering any reliable identification and quantification of individual lipids or lipid classes virtually impossible. The protocols of different tissue extraction and spectrum acquisition techniques will be discussed below.

Beyond tissue extracts, ex vivo NMR spectroscopy of cultured cells and excised tissue will be discussed. Since NMR spectroscopy of perfused cells, and of tissue samples under high-resolution magic-angle spinning (HR-MAS), are domains of research in their own right, the main emphasis of this review will be on NMR spectroscopy of (i) cell suspensions (resembling cell pellets, i.e., without perfusion with cell culture medium) and (ii) static tissue samples (without magic-angle spinning). Both of these NMR analyses require low sample temperatures before and during the NMR acquisition to “freeze” metabolism or, more precisely, to drastically slow down metabolic processes. These processes would otherwise result in erroneous information on the tissue metabolism at the time of harvest. Although these ex vivo techniques are less common than cell perfusion and HR-MAS NMR spectroscopy, respectively, they are of particular interest in cases where (i) only one “snapshot” of a metabolic profile needs to be obtained for each sample, (ii) no time-consuming sample preparation and/or sophisticated sample handling is desired, and (iii) no specialized equipment is available for spectrum acquisition, because probes and NMR tubes commonly used in routine analytical NMR spectroscopy are entirely adequate.

In addition, ex vivo NMR experiments on whole organs have been published, with or without perfusion with medium. The results of these investigations allow one to study metabolism of organs under conditions that are rather close to those dominating in vivo, albeit without constraints imposed by body motion, anesthesia effects [3,4], etc. In addition, monitoring transplantation organs is another application of metabolic whole-organ spectroscopy. However, a detailed discussion of NMR spectroscopy of perfused organs and the study of perfused brain slices are beyond the scope of this review.

This review is subdivided into two parts. The first part (the main body of this article) essentially deals with particular (mostly recent) developments for metabolic ex vivo NMR spectroscopy and is structured by methodology. This includes an analysis of different sample types, such as cell suspensions and spheroids, and metabolite extracts of tissues and cultured cells. In addition, we will also discuss sample preparation procedures and briefly mention a few NMR studies based on isotope-labeling that are of particular interest in the context of our review. While raw-data processing and post-processing have a major influence on the quality of NMR spectra of tissue extracts and intact samples, we will not treat this topic here in great detail since these issues are not specific to metabolic in vitro NMR spectroscopy. Suffice it to say that, in general, the application of appropriate resolution enhancement techniques such as Lorentzian–Gaussian line shape transformation can be beneficial for identification and quantitation of NMR resonances.

Although, in the past few years, in-cell NMR spectroscopy for structural and dynamic investigation of proteins has gained momentum, we will only briefly report on this approach since our overview is predominantly focused on metabolic NMR spectroscopy. The second part (Supplementary Materials) focuses on particular applications of the ex vivo NMR methods mentioned above to specific biological questions. Here, research on cancer, stem cells and cells from different biofluids such as blood and ascites, cerebrospinal fluid etc. [5,6], is of central importance. Finally, some more peripheral applications of metabolic ex vivo NMR will be briefly presented, such as studies on bacteria, yeast, and plant cells. In this overview, emphasis is not only placed on the most recent applications. A number of interesting developments that took place 10 to 20 (or more) years ago proved to be valid and successful methods, although they may not have been followed up by a significant number of applications in the recent past. Thus, short-term impact was not the only criterion for inclusion in this review. However, throughout this review, common and uncommon pitfalls will be highlighted that significantly limit the value of several published methods, recent or not-so-recent, in the research areas covered here.

## 2. Developments for Ex-Vivo NMR Spectroscopy

### 2.1. Techniques for Studying the Metabolism of Cells in Single-Cell Suspension, and of Associated Cells

#### 2.1.1. Study of Phosphorylated Compounds in Treated Isolated Cells

The most commonly used nuclei to be discussed in this review are ^1^H, ^31^P and ^13^C. An interesting example of combined ^1^H- and ^31^P-NMR spectroscopy has been presented recently by Abramov et al. [7]. These authors studied cultured cells obtained from peritoneal effusions (accumulation of body fluid in the abdomen) of patients with ovarian tumors, along with ovarian tissue from the same patients. Here, the aim was to characterize the metabolic patterns of malignant vs. benign disease. The investigation of cancerous cells isolated from peritoneal effusions allowed the characterization and monitoring of metabolic changes occurring in ovarian cancer cells in response to in vitro chemotherapy with paclitaxel (taxol), cisplatin or carboplatin. The authors emphasized that embedding cells in gel threads facilitated continuous monitoring of spectra following incubation with these anti-mitotic drugs; however, they did not describe how they maintained cells during continuous monitoring (by perfusion with oxygenated culture medium at 37 °C?), nor did they present time course data derived from continuous monitoring. Nonetheless, the authors were able to demonstrate that drug (taxol) treatment had similar effects on phosphodiester and UDP-hexose levels (relative decreases normalized to ATP levels), (i) whether cells were incubated as a monolayer in the presence of the drug, before extraction with perchloric acid (PCA) and ^31^P-NMR analysis; or (ii) whether they were drug-incubated and analyzed as intact cells within the 3D gel matrix (single spectrum rather than time course; no data provided for cisplatin or carboplatin effects on gel-embedded cells). The gel used was based on a solubilized basement membrane preparation (Matrigel) mostly made up of laminin and collagen IV. Matrigel is known to not only provide mechanical support for cells, but also to permit cell proliferation when perfused with appropriate culture medium [8,9]; therefore, the value of the data presented critically depends on whether cells were perfused during the NMR experiment. Apart from this issue, a point of concern in this study is the fact that monolayer cell extract data and the gel-embedded whole-cell data were obtained with different clones of cells from peritoneal effusion. Another point of concern is the ambiguous use of the term “cell extract” that appears to equally refer to extracts of isolated cells, and to extracts of excised tumor tissue (the latter should simply be termed “tissue extracts”; both techniques will be discussed in more detail in Section 2.4 and Section 2.5 below, and in Supplementary Materials).

#### 2.1.2. Mobile Lipids Studied in Isolated Intact Cells

^1^H- and ^31^P-NMR spectroscopy of suspended cells has been performed in the context of cisplatin-resistance of a human adenocarcinoma cell line [10]. This work was primarily aimed at studying the effects of multidrug resistance on membrane (phospho)lipids and their metabolites. For ^1^H-NMR spectroscopy, cultured cells treated with cisplatin were rinsed with phosphate-buffered saline (PBS), trypsinized, pelleted, and resuspended in PBS with D_2_O instead of water as solvent. For ^31^P-NMR spectroscopy, cells were resuspended in cell culture medium, with percoll added to prevent cell sedimentation during the NMR experiment. All experiments were conducted at room temperature (^31^P-NMR) or at 37 °C (^1^H-NMR). The latter temperature allowed the detection of lipid methyl and methylene signals because lipids were kept approximately as (semi)fluid as they are under physiological conditions. However, the authors did not comment on the metabolic consequences of keeping the cell suspension at this temperature in the NMR tube without providing the cells with fresh oxygen and other nutrients, and without removing catabolites. Under these conditions, cells will quickly (within one or several minutes) become hypoxic and, consequently, convert most intracellular glucose to lactate, followed by other metabolic artifacts induced by hypoxia. Thus, the relatively broad lipid methylene peak in ^1^H-NMR spectra may overlap with a lactate methyl peak at roughly the same chemical shift; these two signals cannot be distinguished. Nonetheless, the authors exclusively interpreted this peak as stemming from lipid methylene, which does not seem to be justified. In addition, there is no clear distinction between membrane lipids in general and phospholipids (PLs) in particular in this paper; the possibility of intracellular (unpolar) lipid droplets being at the origin of the methyl and methylene peaks is not even mentioned (see also following paragraph). Moreover, differences in other metabolite levels quantified in this work are interpreted in terms of differences in cellular biology between cisplatin-sensitive and cisplatin-resistant cells (with only the former undergoing apoptosis upon treatment), while any artifacts due to hypoxia or nutrient depletion were given no consideration here. When discussing ^31^P-NMR spectra, the authors interpret the phosphomonoester (PME) peaks detected as representing the PL metabolites, phosphorylcholine (PC) and phosphorylethanolamine (PE), which is plausible. However, they also interpret the line width of these overlapping PC and PE signals as indicating the mobility of PL head groups, although the phosphate moiety of a PL head group is diesterified as opposed to that of PC or PE. If there was any phosphorus signal from PL head groups detectable, it would appear as a very broad hump peaking in the phosphodiester (PDE) region (see discussion of Shestov et al. below), not in the spectral region of PMEs. As a consequence, this work is of very limited value due to the methodological issues outlined.

The problem of the intracellular origin of lipid methylene and methyl signals in ^1^H-NMR spectra of rat glioma cell suspensions has been addressed in a very meaningful way over 20 years ago by I. Barba et al. [11]. These authors found a convincing correlation between the “mobile-lipid” (ML) resonance at about 1.24 ppm (methylene) in the spectra, and intracellular lipid droplets identified by fluorescence microscopy analysis (cells stained with the lipophilic dye Nile red). For cells grown at physiological pH, both methods detected ML almost exclusively in cells beyond confluence, whereas they were almost absent in log-phase cells. When grown under conditions of acidic stress (pH 6.2), the ML resonance as well as Nile red-detected lipid droplets were increased even in log-phase. These NMR experiments were conducted with a spin-echo sequence (136 ms echo time) that inverted the lactate methyl doublet, but not the lipid methylene peak of nearly identical chemical shift. This echo time is equal to 1/^3^*J*, where ^3^*J* is the CH-CH_3_ proton-proton scalar coupling constant of the methyl doublet. Thus, the use of a spin-echo sequence to invert the resonance of a coupled spin has to be carefully calibrated as a function of the coupling constant in question. In this way, the authors were able to safely assign the signal in question to ML rather than lactate, in contrast to the work discussed in the preceding paragraph. Thus, the conclusion that intracellular accumulation of ML is a consequence of impaired proliferation (due to either confluence or acidosis) appears to be justified because confounding factors were ruled out in this study.

#### 2.1.3. Investigation of Intact Cells Grown in Spheroids

As shown above, metabolic NMR spectroscopy on whole cells can be performed based on single-cell suspensions, cells embedded in gel (threads), or cells attached to the surface of micro-carriers. In these cases, cells are either separated from each other, or form one-dimensional (gel polymer chains) or two-dimensional (micro-carrier beads) surfaces where they grow in monolayers. However, these cellular environments do not represent the situation found in most mammalian tissues, where cells typically grow forming three-dimensional structures. Therefore, some researchers prefer the use of spheroids, i.e., more or less round-shaped agglomerations of a large number of cells. For instance, Kunz-Schughart et al. presented a ^31^P-NMR spectroscopic study on the metabolism of transformed fibroblasts, where the cell spheroids were contained in a 10 mm diameter tube perfused with a supplemented, phosphate-free medium to maintain cell viability [12]. A special tube design permitted the acquisition of 1 h spectra with a signal-to-noise ratio of 10 using only 2–3 × 10^7^ cells. Hand-selected groups of spheroids were transferred to the perfusion chamber before measurement; this permits control of spheroid size (diameters between 400 and 1400 μm) at the beginning of the experiment. It was shown that a number of ^31^P-NMR-derived metabolic parameters depended on spheroid size. This primarily concerns the levels of PL metabolites, such as the values for PC/cell, PC/PE, and GPC/GPE (glycerophosphocholine/glycerophosphoethanolamine), but also NTP/cell, which were all correlated with an increased G_1_/G_0_ phase cell fraction. By contrast, the energetic status, NTP/P_i_, and pH_i_ (intracellular pH as determined via the chemical shift of the P_i_ peak) showed hardly any dependence on spheroid size. It was concluded that cells in the inner, oxygen-deficient zones of the spheroids appear to adapt their energy metabolism to the environmental conditions before they undergo necrotic destruction as the spheroid size increases. Therefore, the three-dimensional cell spheroids must be considered as a valid alternative to zero, one or two-dimensional cell systems. This is particularly true in cancer research since oxygen-deficient zones are very common in malignant tumors, and can easily be mimicked as a function of spheroid size. Palma et al. presented a comparison of ^1^H-NMR spectra of breast MSF-7 tumor cells grown as a 2D monolayer, as spheroid suspensions, and from single spheroids [13]. They found that fatty acid and glutamine signals were enhanced in spheroids vs. monolayers. High lipid (methylene) peaks have long been known to be characteristic of in vivo tumor ^1^H spectra; thus, spheroids appear to represent these in vivo hallmarks more truthfully than monolayer cultures. Since developments for NMR spectroscopy of perfused cells are an entire research domain in its own right, they are not presented here in detail.

#### 2.1.4. Multinuclear Live Cell NMR Spectroscopy

Beyond metabolic investigations, there is an NMR spectroscopic technique that is applicable to intact cells but not directly related to studying cell metabolism. This very recent method is known as “in-cell NMR”, “on-cell NMR”, or, more generally, as “live cell NMR”, and relates to investigations of structure and dynamics of intracellular and cell-surface macromolecules. These molecules essentially comprise proteins, nucleic acids, glycans, and lipids. High-resolution live cell NMR spectroscopy is commonly performed using isotopic labeling to make particular signals from these biomolecules stand out in spectra of unlabeled compounds, or to render these molecules detectable by replacing NMR-inactive nuclei by NMR-active nuclei [14]. In this way, the NMR-active isotope enables selective detection of structural and functional properties of labelled biomolecules against the backdrop of all unlabeled cellular components. One sample preparation strategy, applied to in-cell protein NMR, consists in inducing protein overexpression in cells (first performed in human cells by Banci et al. [15]). Here, transient protein overexpression and isotopic labeling in human embryonic kidney HEK293T cells was driven by the cytomegalovirus (CMV) promoter. Generally, there is a large variety of isotope-labelled precursors that can be used; for instance, ^13^C-labelled amino acids are common precursors for protein synthesis. In prokaryotes, isotopic labeling of recombinant proteins is usually achieved by growing bacteria (e.g., *Escherichia coli*) in media containing ^15^N-ammonium chloride, ^13^C-glucose, or D_2_O as the only source of metabolic precursors [14]. Upon induction of recombinant protein expression, ^15^N-, ^13^C-, or ^2^H-isotopes are incorporated into the newly synthesized polypeptides, and are readily detected by high-resolution NMR spectroscopy of these nuclei. In the past few years, many sophisticated variants of this approach were developed [16]. Although, in these experiments, labeled NMR-active metabolic precursors are employed, it is not labeled metabolites that are of interest in live cell NMR, but the macromolecules synthesized from these. For this reason, in-cell and on-cell NMR will not be further discussed here.

### 2.2. Ex Vivo and In Vitro vs. In Vivo NMR Spectroscopy in the Context of Metabolomics

#### 2.2.1. NMR Spectroscopy vs. Mass Spectrometry of Cell Extracts

The field of metabolomics has become an important domain of NMR spectroscopy over the past 20 years [17]. This fact has given rise to several publications pointing out the advantages and disadvantages of ex vivo, in vitro, and in vivo NMR spectroscopy in metabolomics research and application. What all three methods have in common is the relatively low sensitivity of NMR methods in general, when compared to the one dominating method in metabolomic analysis, which is mass spectrometry (MS). In fact, Chung et al. began their review of NMR methods in metabolomics with a brief comparison with MS [18], and listed a number of “compensating advantages” of NMR spectroscopic vs. MS. Among these, the ease of sample preparation is noticeable, albeit primarily for HR-MAS; but also sample preparation for NMR spectroscopy of tissue extracts requires less work since no derivatization of the compounds to be measured (to render these volatile for GC-MS) is needed. Another advantage of NMR vs. MS is that in general, no chromatographic metabolite separation, e.g., by LC (liquid chromatography) or GC (gas chromatography), is needed. However, identification and quantification of metabolites with signals in crowded spectral regions of ^1^H-NMR spectra of tissue extracts may benefit from hyphenated NMR, e.g., LC-NMR spectroscopy. Furthermore, metabolites need to be ionized for MS analysis, but not for NMR analysis. Consequently, complex calibration procedures with multiple standard compounds need to be applied for accurate quantitation based on MS spectra because ionization efficiency varies between different molecules. By contrast, with adequate experimental parameters, there is a linear relationship between NMR signal intensities and amounts of compound measured. This means that only one standard compound with known concentration is required for absolute quantitation of all compounds found in an NMR spectrum. In addition to generating more workload, derivatization, ionization, and complex calibration procedures are sources of quantitation errors, which renders NMR spectroscopy both more accurate and precise. Thus, although ex vivo NMR methods are usually able to quantify a metabolic profile of only a few tens of intermediate or high-concentration metabolites (albeit more than in vivo NMR methods), they provide great precision and reproducibility. This advantage makes them particularly amenable to studies in which the metabolome of a cell or organism is perturbed and then compared with the original metabolome [18]. Ideally, ex vivo NMR and MS methods can be combined to benefit from the specific advantages of both methods.

#### 2.2.2. In Vitro vs. In Vivo NMR Spectroscopic Analysis

**Figure 1:**
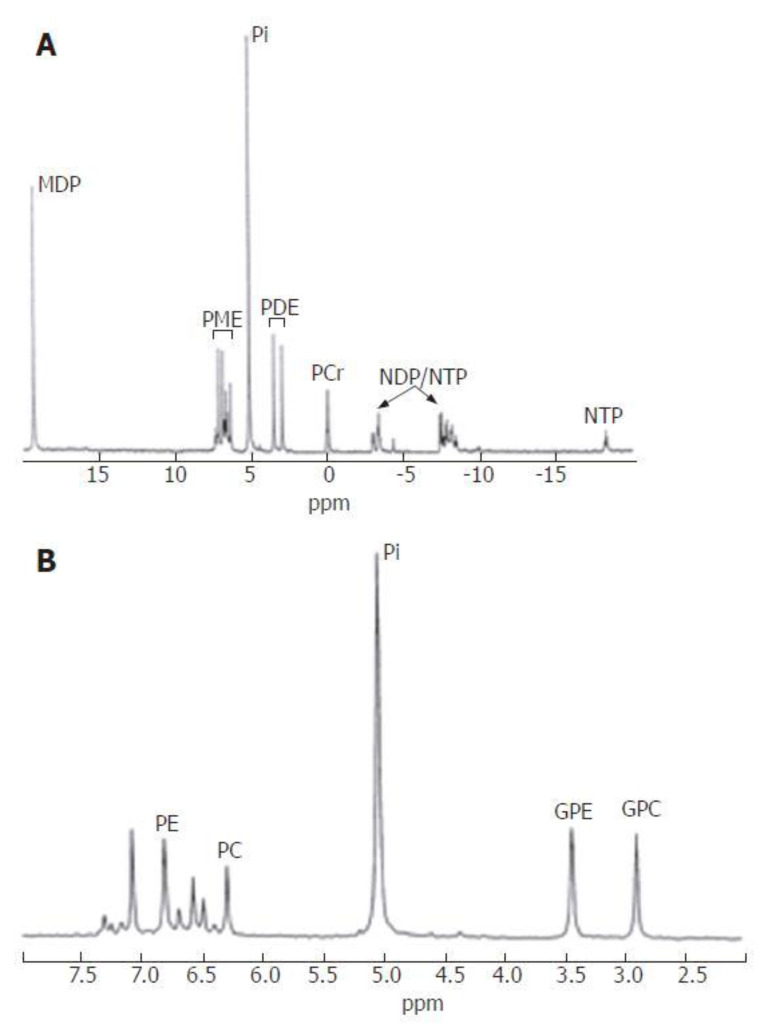
Typical proton-decoupled in vitro ^31^P-NMR spectrum of perchloric acid-extracted normal liver tissue. (**A**): Full spectrum; (**B**): PME and PDE regions. NDP: nucleotide diphosphates; MDP: methylene diphosphonate (chemical-shift reference compound); other abbreviations: see text. Reprinted under the Creative Commons Attribution License (CCAL) terms from [20].

The increase in ^1^H-NMR spectral resolution when going from in vivo NMR to HR-MAS NMR to tissue extract NMR, has been highlighted by Chung et al. [18]. It is obvious that increased resolution permits detection and quantitation of an increased number of metabolites. In addition, HR-MAS and extract spectra can be acquired at significantly higher magnetic fields (high-resolution NMR spectrometers) than in vivo spectra (whole-body imagers/spectrometers), which further improves sensitivity and quantitative accuracy of the experiment. Substantially improved resolution was also found for ^31^P-NMR spectra obtained by HR-MAS vs. in vivo NMR [19], but it is extremely difficult to maintain the bioenergetic status in a tissue sample during sample preparation for HR-MAS. This is a consequence of hypoxia or anoxia subsequent to tissue harvest, i.e., as soon as blood perfusion is interrupted when crucial blood vessels are cut, resulting in ischemia. This means that high-energy metabolites such as ATP will have greatly decreased or even become undetectable by the time the HR-MAS NMR spectrum is acquired. For this reason, authors tend to show only the PME and PDE regions in their published HR-MAS ^31^P-NMR spectra [19]. Such metabolic artifacts can be mostly avoided by quickly freezing the sample upon harvest and extracting the metabolites at low temperatures where enzymatic reactions that could degrade the bioenergetic status are virtually frozen. This has been shown by Cox et al. [20] for liver tissue; although the authors have chosen to present, besides in vivo ^1^H- and ^31^P-NMR liver spectra, in vitro ^1^H-NMR spectra from intact tissue acquired under HR-MAS, they show in vitro ^31^P-NMR spectra (including the NTP region) based on tissue extracts (Figure 1) and not on HR-MAS samples. Moreover, metabolic conditions altered during sample preparation can also affect processes other than energy metabolism, for instance glycolysis. For this reason, ex vivo ^1^H-NMR spectra from intact tissue samples usually exhibit particularly intense lactate resonances, which are to a great extent artifacts introduced by anaerobic glycolysis induced by a lack of oxygen, the latter being the result of ischemia. This is true notwithstanding that many tumors typically produce large amounts of lactate in vivo [21,22]. Tumor-generated lactate would also be detectable in excised tissue and in spheroids, but undetectable in cells harvested and/or extracted from monolayer cultures because lactate produced by tumor cells is often efficiently exported into the extracellular space (see [23] for ^1^H-NMR spectroscopy of extra vs. intracellular metabolism). Overall, ex vivo ^1^H-NMR spectra measured under these circumstances must be interpreted with utmost caution, even though there may be reasonably good correlations for particular ex vivo HR-MAS and in vivo NMR peak ratios such as (choline + creatine + spermine)/citrate in prostate cancer [24].

A review by Whitehead et al. [25] cited work by Sitter et al. [26] stating that in ^1^H HR-MAS spectra of breast cancer tissue, lactate was found to be a minor component, whereas in the corresponding PCA extract spectra lactate was more prominent. This seems to contradict the statement on lactate signals made in the preceding paragraph. However, one should consider that (i) the “prominence” of a given peak is not a quantitative measure of its intensity. Variations in line widths for different peaks can have a major influence on which peaks appears to be “prominent”. For instance, a signal of any given intensity (i.e., integral, or area under the peak, representing a given amount of spins) from small, highly mobile molecules, such as the lactate methyl resonance, may become extremely narrow (and, therefore, high) when observed in an extract, compared to the corresponding peak in an HR-MAS spectrum, and may therefore appear very “prominent”. On the other hand, a signal stemming from a larger, less mobile molecule, or from a molecule that undergoes exchange with another compound at an intermediate exchange rate, will experience less narrowing in an extract and, therefore, appear less “prominent”. In addition, as stated above, (ii) sample handling during the sample preparation period has a major influence on the occurrence of anaerobic glycolysis, which may also affect lactate signal comparisons between HR-MAS and extract spectra. Further details concerning the appearance of tissue extract spectra are presented below in Section 2.4 and Section 2.5.

Recently, Chandra et al. addressed the issues of sensitivity and specificity in in vitro NMR spectroscopy by suggesting new methodological developments [27,28]. Significantly improved sensitivity was reported by combining two previously developed techniques, i.e., (a) controlled detuning of the probe such that the “spin noise tuning optimum” (SNTO) was obtained, and (b) using an externally cylindrical NMR tube made of susceptibility-matched glass, containing a slot-like (non-cylindrical) sample space, instead of a conventional 5 mm NMR tube. In this design, the main horizontal axis of the sample chamber within the sensitive volume of the transmitter/receiver coil, is in parallel with the electric B_1_ field of the (saddle) coil. Here, no sample is present in the region most susceptible to the generation of spin noise [27]. The authors also developed a tailored sequence to optimally identify metabolites whose signals are usually hidden under broad lipid and protein signals. This sequence consists of a suite of selective and non-selective CPMG-filtered 1D and 2D TOCSY/HSQC experiments [28].

### 2.3. Methodological Aspects of NMR Experiments with ^13^C Labels Metabolized by Perfused Isolated Organs and Perfused Cultured Cells

Nath et al. [29] have presented ^13^C glucose labeling studies using 2D NMR of perfusate for investigating ex vivo whole-organ (kidney) metabolism. Their aim was to assess whether metabolic-pathway activity within a machine-perfused porcine kidney can be determined using ^13^C/^1^H heteronuclear single quantum coherence (HSQC) NMR spectroscopy of the perfusate during hypothermic machine perfusion (HMP), and of kidney cortex extracts after HMP. The authors used a perfusion medium containing a physiologically relevant amount (10 mM) of [U-^13^C]glucose. However, in this work, the term ‘tracer’ is used in clear contradiction with its actual meaning based on the well-established and uncontroversial tracer theory [30], and, therefore, should have been avoided. The notion ‘tracer’ actually designates a labeled molecule that is added to a (native) biological organism or system at a concentration that is very small compared to that of the corresponding natural, unlabeled compound (‘tracee’), as to not significantly perturb the native metabolism of the tracee [30]. Under these circumstances, observation of the tracer allows one to identify the metabolic fate that the much more concentrated tracee experiences at the same time. This is certainly a different type of experiment than the one described by Nath et al. (the same statement applies to the second ^13^C-NMR experiment described below). Since in this case all glucose in the perfusion medium is ^13^C-labelled, we have here the curious situation that ‘tracer’ and ‘tracee’ would designate the very same molecules, which is certainly illogical in terms of the very concept of the tracer theory [30]. What is referred to in these papers is simply labeled-isotope experiments.

The authors were able to demonstrate the presence of active glucose metabolism even under conditions of (i) low temperature (4 °C) prevailing under HMP, and (ii) the circulation of an artificial perfusion fluid instead of blood. This was shown by the detection of labeled metabolites in the extracellular perfusion fluid within 6 h, hinting at cellular uptake of glucose, glycolysis and transport/efflux of resulting metabolites, all occurring within this time period. However, the proportion of de novo metabolites was modest (less than 15% ^13^C enrichment for lactate in perfusion fluid, and markedly less for other labeled metabolites). In addition, quantitating a variety of ^13^C-labelled glucose metabolites and their ^13^C enrichment, Nath et al. also quantum-mechanically modeled multiplets originating from ^13^C-^13^C couplings in the most prominent ^13^C-labelled glucose metabolites, and compared the latter with measured spectra. The line shapes used for this purpose were cross sections through the middle of the 2D ^1^H/^13^C HSQC spectra, each cross section obtained for the ^1^H chemical-shift value corresponding to the peak maximum. Although the authors mentioned that these simulations can form the base for a subsequent isotopomer/isotopologue analysis, they did not provide such a complete analysis. It should be noted that in the past decades, a very large body of metabolic ^13^C-NMR-related research has been performed, in different organs and using a variety of experimental protocols, including also hyperpolarization experiments and isotopomer analysis [31,32,33,34,35,36,37,38]. However, this field of research is vast and clearly deserves a review of its own.

More recently, Shestov et al. presented a very interesting ^13^C-NMR study on melanoma cells perfused in a bioreactor [39] system. To avoid artifacts stemming from a concentration increase for glucose or glutamine due to addition of ^13^C-labelled substrates, they determined glucose and glutamine levels in the perfusion medium during the experiment, and regulated the continuous infusion of these ^13^C-labelled substrates such that stable standard concentrations were ensured. ^13^C enrichment in metabolites of ^13^C-labelled glucose and glutamine was not only observed in ^13^C-NMR spectra, but also in ^1^H-NMR spectra of the cells, thanks to the large ^13^C-^1^H ^1^*J* coupling constants (about 124 Hz). By contrast, the resolution of ^31^P-NMR spectra of intact cells was not sufficient to detect any ^13^C-^31^P ^2^*J* coupling (small coupling constants, about 4 to 7 Hz for phosphorylated metabolites of interest). These coupling patterns can only be detected through high-resolution ^31^P-NMR spectroscopy of cell extracts, i.e., in homogeneous solutions of metabolites (see Supplementary Materials Section SI 1.1.1, ) [40,41]. Likewise, ^13^C-^31^P ^2^*J* coupling of these compounds is equally undetectable in ^13^C-NMR spectra obtained from whole cells, as opposed to cell extracts.

### 2.4. Focus on Extracts of Body Tissue and Cultured Cells in NMR Spectroscopy

#### 2.4.1. Basic Tissue Extraction

Some advantages and disadvantages of using tissue extracts, as opposed to whole cells or excised tissue, for in vitro metabolic NMR spectroscopic analysis are presented above in the Introduction section, and in Section 2.2. The basic principles of extracting tissue for NMR are described abundantly in the scientific literature (see Chung et al. for a recent review [18]). Briefly, biological tissue or cultured cells are prepared by extraction with a liquid that denatures the tissue proteins that precipitate for the most part. This inactivates enzymes that would otherwise, within a few seconds, lead to uncontrolled metabolism as soon as cells are exposed to a non-standard environment following harvest (i.e., after tissue excision from the body, or cell removal from the culture flask). As a consequence, the metabolic makeup of the extract would be influenced by extraction artifacts and yield erroneous metabolite concentrations. Since metabolite extraction always includes crushing cells and organelles, this homogenization process releases intracellular metabolites into solution. In addition, it also releases metabolites that were bound to proteins prior to denaturation, and that are essentially undetectable by in vivo NMR spectroscopy, or by in vitro NMR based on intact cells. The precipitated proteins are spun down and the supernatant containing the metabolites is further processed. A brief overview of basic extraction procedures is given below; a step-by-step presentation of extraction protocols can be found in Table S1 (Supplementary Materials).

If only water-soluble metabolites are of interest, the extraction liquid of choice is 5% perchloric acid (PCA). In this case, the resulting metabolite solution needs to be brought to a well-defined (approximately neutral) pH after proteins are removed. This is preferably done with potassium hydroxide because the resulting potassium perchlorate is not soluble in water in large quantities (solubility at 25 °C: 2% of solute mass, as opposed to sodium perchlorate (67%)). In this way, high salt concentrations in the extract are prevented that might otherwise hamper NMR spectroscopy because the sensitivity of NMR probes can be substantially reduced by the electrical noise generated by conductive samples. Electrical conductivity is a function of salt concentration and mobility of the ions in solution. Once the precipitated KClO_4_ is spun down, the supernatant is freeze-dried, and the dried metabolites are re-dissolved in D_2_O (deuterated water) for analysis by ^1^H-NMR after pH adjustment. Lyophilization serves to prevent the huge proton signal from water (about 110 M water proton concentration) from rendering the detection and quantitation of signals from metabolites (≤mM concentrations) difficult or impossible. If only heteronuclei are to be used in NMR analysis (^31^P, ^13^C, ^19^F, etc.), H_2_O can be used as a solvent. An external standard is normally added to the extract to permit chemical-shift referencing and quantification, although the use of internal standards is also possible.

**Figure 2:**
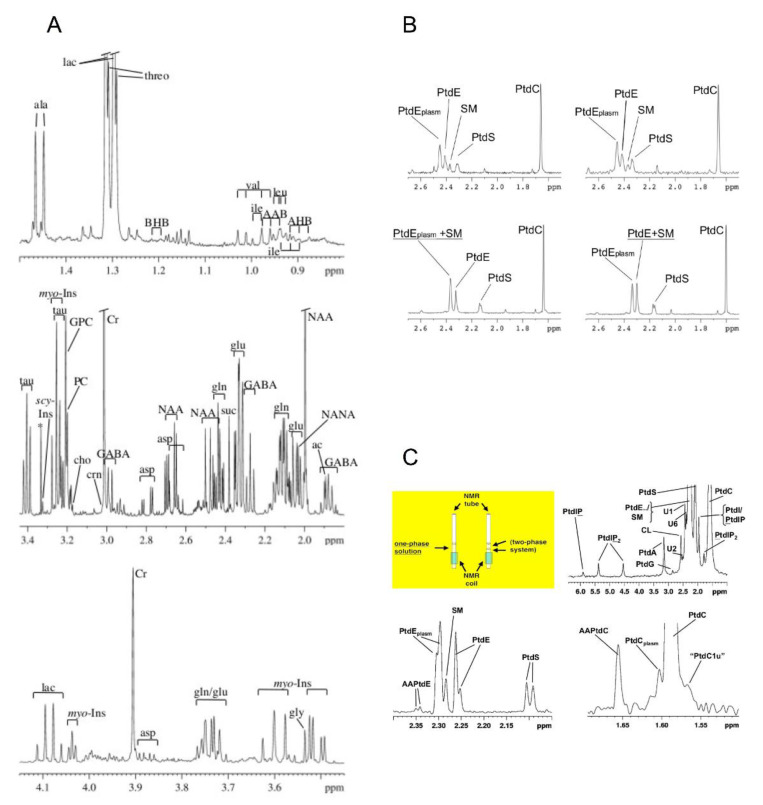
^1^H-NMR spectroscopy (400 MHz, 9.4 T) of the aqueous phase of a brain tissue extract from a female Lewis rat. Panel (**A**). Subregions of a typical ^1^H-NMR spectrum (400 MHz, 9.4 T) of the aqueous phase of a brain tissue extract from a female Lewis rat. All three panels demonstrate the extremely high resolution obtainable using the protocol presented in the paper cited below. Neither chelating agent nor ion exchange resin has been used during sample preparation. Center and bottom spectra show the existence of many unassigned low-intensity peaks that hint at the huge dynamic range covered by high-resolution ^1^H-NMR spectroscopy of tissue extracts if performed using optimized experimental parameters. These weak but well detectable signals can potentially be identified and quantified in the future. Several detected metabolites are specific of brain tissue, e.g., the neuron marker NAA or the neurotransmitter GABA; however, most compounds are involved in a broad spectrum of metabolic pathways that are common to mammalian cells, such as amino acid, branched-chain organic acid, polyol, (phospho)lipid and energy metabolism as well as in glycolysis and glutaminolysis, and in functions such as osmoregulation, cell growth and proliferation. The asterisk denotes the methyl resonance stemming from a methanol impurity. Abbreviations: ala, alanine; lac, lactate; threo, threonine; BHB, β-hydroxybutyrate; val, valine; ile, isoleucine; leu, leu-cine; AAB, α-aminobutyrate; AHB, α-hydroxybutyrate; tau, taurine; *scy*-Ins, *scyllo*-inositol; *myo*-Ins, *myo*-inositol; crn, creatinine; Cr, creatine; asp, aspartate; gln, glutamine; glu, glutamate; suc, succinate; NANA, *N*-acetylneuraminate; ac, acetate; gly, glycine; *, residual methanol from extraction solvent; for other abbreviations: see text. The small peaks at the base of the ala doublet stem from the lactate ^13^C satellite doublet (top). Panel (**B**). Phosphatidylethanolamine regions of phospholipid ^31^P-NMR spectra (162 MHz, 9.4 T) of brain tissue extracts from female Lewis rats. (Top left) Brain tissue concentration, 236 mg/mL; CDTA concen-tration and pH in the aqueous component of the solvent, 200 mM and 7.33, respectively; measurement temperature, 297 K. PtdE_plasm_ and SM signals are well resolved. (Bottom left) Brain tissue concentration, 236 mg/mL; CDTA concentration and pH in the aqueous component of the solvent, 1000 mM and 7.36, respectively; measurement temperature, 297 K. PtdE_plasm_ and SM signals overlap entirely; they cannot be resolved despite reduced line width, compared with the top left spectrum. (Top right) Brain tissue concentration, 118 mg/mL; CDTA concentration and pH in the aqueous component of the solvent, 50 mM and 7.14, respectively; measurement temperature, 297 K. PtdE and SM signals are well-resolved. (Bottom right) Brain tissue concentration, 118 mg/mL; CDTA concentration and pH in the aqueous component of the solvent, 50 mM and 7.14, respectively; measurement temperature, 277 K. PtdE and SM signals overlap entirely; they cannot be resolved, despite reduced line width compared with the top right spectrum. Abbreviations: PtdE_plasm_, ethanolamine plasmalogen; PtdE, phosphatidylethanolamine; SM, sphingomyelin; PtdS, phosphatidylserine; PtdC, phosphatidylcholine. Panel (**C**). Phospholipid ^31^P-NMR spectroscopy (162 MHz, 9.4 T) of brain tissue extracts from female Lewis rats. (Top left) A one-phase system (left) was preferred over a two-phase system (right). The commonly used two-phase system hampers correct PL quantitation because most or all of the upper phase is located outside the sensitive volume of the coil. (top right) Complete ^31^P-NMR PL spectrum of rat brain. For better visibility of weak signals (PtdIP, PtdG), exponential line broadening (LB = 3 Hz) was applied. In this representation, several PL signals are not well-resolved, notably in the PtdC and PtdE regions. For PLs generating more than one ^31^P-NMR signal, observed nuclei are underlined (PtdIP, PtdIP, PtdIP_2_, PtdIP_2_). Currently, unassigned signals are denoted by “U_n_” (where n = 1, 2, ...). (**Bottom left**) PtdE and PtdS regions of the same spectrum. For better peak resolution, Lorentzian–Gaussian line shape transformation was applied (LB = −1 Hz, GB = 0.3). Because of these processing parameters, many very weak PL signals are difficult to detect. However, at least two peaks can be discerned for each PtdE, PtdE_plasm_, AAPtdE, and PtdS. (**Bottom right**) PtdC region obtained with the same processing parameters as the PtdE region. Several signals at the base of the dominating PtdC resonance were detected unambiguously, while they cannot be discerned in the upper spectrum generated with exponential line broadening (AAPtdC, PtdC_plasm_, PtdC_1u_). In addition, the currently unassigned PtdC analog, PtdC_1u_, further minor resonances may be present upfield from PtdC. Abbreviations: PtdIP, phosphatidylinositol phosphate; PtdIP_2_, phosphatidylinositol diphosphate; PtdA, phosphatidic acid; PtdG, phosphatidylglycerol; CL, cardiolipin; PtdE, sum of PtdE, PtdE_plasm_ and AAPtdE; PtdI, phosphatidylinositol. For further abbreviations see legend to panel B. This figure is adapted from [42] with permission.

If only lipids are of interest, the extraction liquid of choice may be a combination of methanol and chloroform (i.e., the well-established solvent used for Folch extraction). However, in many cases both lipids and water-soluble metabolites are of interest. In this case, tissues will first be homogenized in methanol which denatures most proteins; then, chloroform will be added to this mix, and the resulting mixture will be vortexed. Subsequently, water will be added and the resulting mixture will be vortexed again, upon which two distinct phases will form. The solvent of the upper phase consists of methanol and water; the solvent of the lower phase consists of chloroform; and the precipitated proteins (and other insoluble compounds) form the interface between those two phases. This separation process can be accelerated and completed by centrifugation, and the two phases are processed separately for NMR spectroscopy:

- The lipid-containing phase is recovered and the solvent (chloroform/methanol) evaporated under a nitrogen stream. The dried lipids are then redissolved, either (i) in deuterated chloroform for ^1^H-NMR analysis of all lipids; or (ii) in a well-defined mixture based on methanol, chloroform and water (ideally 40:50:10% *v/v*) for ^31^P-NMR analysis of PLs, based on their polar head groups. Optimization of this solvent mixture is essential for obtaining highly resolved ^31^P-NMR spectra, as the polar head groups of the PLs need to be mobile and free of complexation with cations that have intermediate exchange rates with the phosphate moieties of these head groups. Using more than 10% water will still result in highly resolved ^31^P-NMR spectra, but also in the formation of a layer that mostly consists of water and methanol, and that lies on top of the chloroform/methanol volume containing the PLs (Figure 2C, two-phase system). However, for efficient and accurate absolute PL quantification, the solvent mixture should form one single phase (rather than two phases, only one of which contains the PLs to be analyzed).
- The water/methanol phase that contains the water-soluble metabolites should first be subjected to evaporation of methanol under a nitrogen stream; then, the remaining aqueous solution is to be freeze-dried, and the dried metabolites to redissolved in D_2_O and pH-adjusted. In all cases, external standards for chemical-shift referencing and quantitation are common practice. Detailed protocols for these extraction procedures are given elsewhere [42,43]. One of these references is supplemented with an educational video visualizing the crucial steps of sample processing and spectrum acquisition [42].

#### 2.4.2. Details of Tissue Extraction Techniques and Specialized Methods

##### Speeding Up Tissue Extract Analysis

In addition, the basic, general-purpose techniques mentioned in the preceding section, a number of more specialized extraction methods were developed. We will focus on procedures that are typically used in metabolic in vitro NMR spectroscopy. There are numerous other tissue extraction methods not dealt with here, but many of these are used in MS much more than they are in NMR, e.g., the isopropanol/acetonitrile/water method described by Fiehn et al. [44]. Mili et al. reported a fast and ergonomic extraction method for adherent mammalian cells [45]. This study was motivated by the need to make the extraction of cultured cells (HeLa) amenable to high-throughput analysis. Three steps of the protocol were optimized sequentially; step 1: rinsing the cells to prevent compounds present in the culture medium from attaching to the cell surface before metabolite extraction; step 2: quenching intracellular metabolism to avoid artifacts based on perturbations of intracellular metabolism due to cell handling; and step 3: the actual extraction of metabolites from the cells, including the preparation of NMR samples from the raw extract. In step 1, cells were rinsed with “warm” (temperature not given) phosphate-buffered saline (PBS). However, the authors did not suggest the use of cold PBS, and apparently did not attempt to speed the rinsing process by adding/removing the rinsing fluid to/from the Petri dish by pouring instead of pipetting, akin to a protocol utilized elsewhere (for references, see Section 2.5). This would have enabled 2 rinses to take place in less than 30 s (instead of 60 s) and slowed down metabolism during that time.

Steps 2 and 3 were evaluated together because metabolism quenching and extractions usually occur simultaneously. The authors found that simply extracting cells by rinsing with cold methanol as a solvent yielded, for most but not all metabolites, levels similar to those obtainable through rinses with methanol/water mixtures, with or without scraping the cells into the solvent. The protocol recommended by Mili et al. [45] is certainly simple and fairly fast, but rather problematic if completeness and accuracy of absolute metabolite concentrations are of importance. Further details of their protocol are discussed in Supplementary Materials.

##### Choosing Solvents for Metabolite Extraction

In an older study, Le Belle et al. compared the dual extraction method, mentioned in the preceding paragraphs, with PCA extraction, for rat brain tissue or cultured rat astrocytes being extracted and analyzed by ^1^H-NMR spectroscopy [46]. In addition, in this publication, only water-soluble metabolites were evaluated. Still, these particular authors found dual extraction preferable, for the following reason. When protein and cell debris pellets, spun down after the initial dual or PCA extraction, were re-extracted with methanol/chloroform/water, the metabolite yields from the PCA pellets were consistently greater than those from the corresponding dual-extraction pellets. This indicated a less efficient initial PCA extraction. However, PCA was shown to precipitate cellular proteins more efficiently, which may be advantageous when residual proteins in the final aqueous extract need to be minimized to avoid deformation of the ^1^H-NMR baseline. Although metabolic yields were very similar for the two extraction methods, lactate levels were conspicuously higher in dual-extraction samples than in PCA samples. This may be explained, at least in part, by the less efficient protein precipitation observed in dual extraction, which may have resulted in some residual activity of glycolytic enzymes for the latter extracts. In view of these findings, it seems that overall, PCA with its improved protein denaturation and precipitation has a clear advantage over dual-extraction if only water-soluble metabolites are of interest. In cases where the somewhat lower PCA extraction efficiency is a serious issue, re-extraction of the PCA pellet is to be recommended. As in the preceding paragraph, it has to be mentioned here that any assessment of the dual-extraction method should consider its additional benefit of generating an organic extraction phase that can be used for lipid analysis. By contrast, in PCA extraction, lipids are spun down together with precipitated proteins and other cell debris, and discarded (or used for total protein quantification). 

Another comparison between different extraction solvents was published by Lin et al. [47]. These authors did not evaluate lipid extraction and quantitation either. The following extraction solvents were used: perchloric acid, acetonitrile/water, methanol/water, and methanol/chloroform/water. In addition, the authors tested two different types of pretreatment applied to muscle and liver tissues before extraction: grinding frozen wet or lyophilized tissue under liquid nitrogen, and homogenizing wet tissue without prior freezing (see [48] for other homogenization methods). As in the other works discussed in this and the following sections, only ^1^H-NMR spectroscopy of water-soluble metabolites was utilized. This is most certainly due to the fact that a sizable number of different basic biomolecules can be quantitated within a short period of time by this method, as is desired for routine metabolomics. However, additional phosphorylated compounds would be accessible if ^31^P-NMR was included in the protocol, in particular if the organic solvent phase was also subjected to ^31^P-NMR spectroscopy. The authors observed that the yields of low molecular weight metabolites were similar among the different solvents used for extraction [47]; however, acetonitrile-based extractions provided poorer fractionation and did not prevent extraction of lipids and macromolecules into the polar solvent. Extraction using perchloric acid produced the greatest variation between replicates due to peak shifts in the spectra, while acetonitrile-based extraction produced highest reproducibility. Spectra from extraction of ground wet tissues generated more macromolecules and lower reproducibility compared with other tissue disruption methods. Note that, in this work, no analytical metabolite quantitation was attempted; spectra were only evaluated using principal component analysis (also abbreviated PCA, unfortunately) based on areas integrated within individual spectral bins (segments each corresponding to a bin width of 0.005 ppm). This resulted in principal-component plots (of PC1 vs. PC2) showing data clustering according to the different extraction protocols, and loadings plots for PC1 showing relative contributions of individual peaks to differences between spectra representing these extraction protocols. The authors’ final conclusion was that overall, disregarding metabolite quantitation, methanol/chloroform/water extraction was the method to be preferred when considering both yield and reproducibility of the hydrophilic metabolites as well as recovery of the hydrophobic metabolites.

##### Solvent Removal in Manual and Semiautomatic Extraction Procedures

An often-neglected detail in tissue extract preparation for NMR spectroscopy has been investigated in a rather recent paper by Petrova et al.: effects on metabolite quantitation of the type of solvent removal chosen before re-dissolving the sample for NMR measurement [49]. The authors studied two techniques: speed vacuum centrifugal concentration (SpeedVac), and freeze-drying by sublimation (lyophilization). Three cultured cancer cell lines (MiaPaCa-2, Panc-1, and AsPC-1) were first rinsed three times with cold PBS buffer, and then stored at −80 °C prior to cell extraction. Then, frozen cells were thawed on ice, resuspended in 1.5 mL of ice-cold chloroform-methanol-water (1:1:1) solution and vortexed, after which they were chilled on ice for 15 min. The tubes were then centrifuged for 15 min at 15,000× *g*, and the (hydrophilic) top layer was transferred to an Eppendorf tube for solvent removal by freeze-drying or SpeedVac. 1 mM trimethylsilyl propionate (TSP; for chemical-shift referencing) and 0.01% sodium azide (to prevent growth of germs) were added to 150 mM PBS based on D_2_O (rather than H_2_O) at pH 7.4. The dried samples were then redissolved in this PBS solution and analyzed by 1D and 2D ^1^H NMR spectroscopy at 850 MHz and 298 K. For statistical analysis, each resulting 1D spectrum was divided into 359 manually selected buckets which were used for PCA (principal component analysis) and PLS-DA (partial least squares-discriminant analysis). As in Lin et al.’s work discussed in the preceding paragraph, no quantitation of metabolite concentrations was achieved; statistical analysis was directly performed on 1D ^1^H-NMR spectra, each normalized to total intensity. The authors found that, unsurprisingly, in freeze-dried samples, a number of metabolites were detected that seemed to be absent from SpeedVac samples, but the opposite case was extremely rare (only example identified: trimethylamine in Panc-1 cells). Moreover, the relative levels of many metabolites seemed to depend on whether freeze-drying or SpeedVac was employed for solvent removal prior to NMR sample preparation. Thus, this study confirmed current laboratory practice that generally relies on freeze-drying, not on SpeedVac.

**Figure 3:**
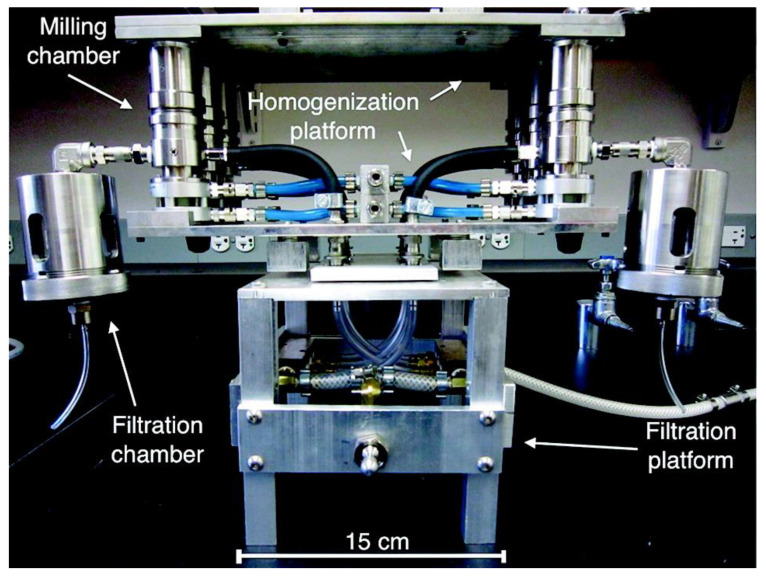
Photograph of the major components of the assembled SAMBED. The air compressor and vibrational shaker are not shown. Reprinted with permission from [50]. 2012, American Chemical Society.

Ellinger et al. suggested a semiautomated extraction method for metabolomics [50]. This project was driven by the intention to (i) reduce manual manipulations that may be a source of error, and (ii) speed up the extraction process. The prototype of their semiautomated metabolite batch extraction device (SAMBED) enables six parallel extractions of samples ranging between 0.05 and 1.0 g tissue each, and integrates the three basic steps involved in the extraction procedure: tissue homogenization, actual metabolite extraction, and sample filtration. This system consists of six integrated components: a milling chamber, a vibrational shaker, a solvent reservoir, a homogenization platform, a filtration chamber, and a filtration platform (Figure 3). Apart from the separation of supernatant from cellular debris and precipitated compounds (filtration instead of centrifugation), also the solvents used differ substantially from established extraction protocols used in ^1^H-NMR metabolic analysis. For instance, the use of a mixture of acetonitrile, methanol and water as an organic extraction solvent appears to be inspired by HPLC analysis. Moreover, aqueous extraction by way of using water at 95 °C is fundamentally different from conventional protocols keeping the sample at low temperatures (4 °C), whenever possible, to suppress chemical reactions that may lead to erroneous results. Moreover, tissue samples need to be dry (lyophilized) before being processed by SAMBED, which adds freeze-drying before the extraction procedure, a step that is not needed in conventional extraction protocols. Thus, although an impressive acceleration of their particular extraction process, based on dried liver tissue, was reported when SAMBED was compared to manual execution of the same procedure, the question of how speed and quality of SAMBED extraction relate to those obtainable by classical extraction methods based on wet tissue has not been addressed. Apart from this question, overall confidence intervals measured for absolute concentrations of all metabolites were comparable between SAMBED and manual extraction procedures.

##### Temperature Control in Preparation of Tissue Extracts for NMR Analysis

The influence of temperature during sample preparation on detection and quantitation of metabolites has been addressed by Haukaas et al. [51]. In this work, the impact of different delays between tissue sampling from the living organism and freezing was determined. Although these authors measured metabolic profiles by HR-MAS of excised tissue rather than by analyzing tissue extracts, their work is directly relevant to extract NMR spectroscopy. No alterations in tissue ^1^H-NMR metabolic profiles of breast cancer xenografts were reported for a delay, at room temperature, of up to 30 min, but profiles changed for longer delays (measured up to 2 h). The authors also reported a general decrease in metabolite signals due to tissue sampling; they compared metabolic profiles of samples measured right after tissue sampling with those frozen in liquid nitrogen right after tissue sampling, and thawed and analyzed at a later time point. These results seem to suggest that once breast cancer tissue has been removed from the living organism and dissected into individual pieces for analysis, it can be left standing at room temperature for up to 30 min without significant metabolic degradation. As a consequence, metabolic profiles obtained by ^1^H-NMR spectroscopy should not be affected by delays of this order of magnitude. However, one would be misguided to believe that the metabolic profiles obtained in this way truthfully represent snapshots of the profiles present in the living tissue before resection. First, particular metabolic processes change within seconds, not minutes, due to induction of hypoxia and nutrient depletion upon cutting blood circulation, as outlined above. In fact, vessels are cut right at the start of surgical tissue removal, while the piece of tissue in question is still physically located in situ. Second, for resections from humans, the speed of tissue removal is limited by precautions that surgeons have to take to protect any normal tissue from unnecessary damage. Third, some more time is needed if the sample is to be subdivided, before freezing, into several pieces for separate analyses, as outlined in Haukaas et al. [51]. Thus, even if tissue is said to be frozen or analyzed “immediately” after sampling, there may be a delay of up to one or several minutes during which time dramatic metabolic changes can occur within the sample, notably those related to anaerobic glycolysis (which possibly also contributed to the high lactate peak in tissue not frozen). There are special freezing methods such as ‘funnel-freezing’ [46] or ‘freeze-blowing’ that result in tissue being frozen in situ before excision [52]; however, these techniques are hardly amenable to application in humans. Furthermore, the results presented in Haukaas et al. [51] are based on areas under peaks (NMR resonances), not on absolute quantities such as mM or mg metabolite per g tissue. Since the spectra were acquired with T_2_-weighting, T_2_ effects may have affected relative signal intensities, in conjunction with T_1_ effects. Such effects may, at least in part, be responsible for the reduction in areas under the peaks observed for metabolites after tissue freezing. This should be kept in mind for comparison with tissue extract studies because the latter often aim for absolute metabolite quantitation.

##### Tissue Extract Studies Complementary to Metabolic Investigations of Effusions and Organ Perfusates

Tissue extract studies have also been performed on ovarian tumors [7]; results based on cells isolated from peritoneal effusions of the same patients are presented above in Section 2.1.1. In this work, ^1^H-NMR spectroscopy was performed on PCA-extracted samples of benign ovarian tumors, and of different types of malignant ovarian tumors (tissues varying in differentiation status; metastases). Between benign tumors, on the one hand, and malignant ovarian tumors in the moderately to poorly differentiated histologic subtypes on the other, the most prominent spectral differences were found for lactate (lac) and total choline compounds (tot.cho). Here, the relative levels of lac and tot.cho, normalized to ATP, were found to be decreased in cancerous vs. benign tissues. However, care should be taken when using these ratios for biochemical interpretations of the underlying metabolism. As pointed out above, lac and ATP levels are prone to metabolic artifacts stemming from tissue excision and handling. Further developments in NMR spectroscopy of cancer cell extracts are presented in Supplementary Materials.

Nath et al., whose analysis of kidney perfusate has been discussed above in Section 2.3, also studied the metabolic profiles of tissue from the kidneys used in the same experiments [29]. They excised sections of the kidney cortex, snap froze these in liquid nitrogen, and pulverized them to a fine powder using a manual cryogrinder. After this, the powdered sample was added to a homogenization tube containing chilled methanol (−80 °C) to quench ongoing metabolism, which was followed by sample homogenization using a homogenizer. Once homogenized, the samples were mixed with water and chloroform, such as to achieve a solvent mixture with a 1:1:1 volume ratio of water:chloroform:methanol, i.e., the classical dual extraction protocol. By analogy with their findings for kidney perfusate (Section 2.3), the appearance of uniformly ^13^C-labelled lactate and alanine in tissue samples demonstrated that de novo glycolysis was occurring in the non-physiological environment created by modifications of kidney perfusion (e.g., temperature, oxygenation, oxygen carriers) under conditions of HMP.

### 2.5. Overview of Techniques for Particular Applications of Metabolic Ex Vivo NMR Spectroscopy

**Figure 4:**
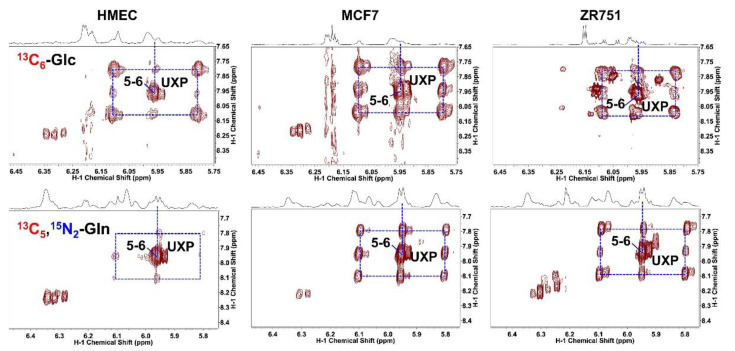
^13^C incorporation from ^13^C_6_-glucose versus ^13^C_5_,^15^N_2_-Gln into pyrimidine nucleotides in three breast cell lines. Cells were grown for 24 h in the presence of 5 mM each labelled glutamine (**top row**) or glucose (**bottom row**) compound. 2D TOCSY spectra were recorded at 600 or 800 MHz (14.1 or 18.8 T, respectively) using a mixing time of 50 ms along. The 2D TOCSY contour maps are shown along with the 1D high-resolution ^1^H spectra. Dashed boxes depict the ^13^C satellites of C_5_ to C_6_ cross-peaks of the uracil ring in UXP with horizontal pairs, vertical pairs, and 4- corner satellites representing ^13^C labeling at C_5_, C_6_, and C_5,6_ of uracil, respectively. Reprinted with permission from [59]. 2017, Elsevier.

The literature presented above in Section 2 clearly indicates that (i) ^1^H is the one nucleus that by far dominates all metabolic NMR spectroscopy of tissue extracts and excised tissue, whereas ^13^C is much less frequently employed (Figure 4). Over the past 20 years, the use of ^31^P has mostly been limited to a few cancer-related research programs, whereas other nuclei appear to be almost inexistent in the domain of metabolic NMR of extracts and excised tissue. Furthermore, (ii) nearly all tissue extract NMR spectroscopy of the past 20 years investigated water-soluble metabolites only, even though many groups use the biphasic (or dual) extraction procedure that collects lipids in an organic phase, while the aqueous phase simultaneously collects water-soluble compounds. To some extent, this situation is due to the fact that ^1^H is the most ubiquitous, and also the most NMR-sensitive nucleus present in biomolecules. Moreover, the new field of metabolomics has put nearly all the emphasis on rapid, high-turnover analysis applicable to a wide range of tissues, biofluids and biological problems, potentially amenable to automation and industrial routine application. Another reason is that for detailed lipid analysis, highly sensitive methods exist that routinely include a separation step, such as mass spectrometry preceded by liquid chromatography (LC-MS). LC-MS of lipids is well established and superior to ^1^H NMR since lipid spectra produced by the latter method exhibit many overlapping peaks from different lipid molecules, limiting analysis to a few weakly resolved signals representing entire classes of lipids. However, there is a number of special applications and scientific questions where heteronuclear NMR spectroscopy can indeed provide valuable information in the study of extracts and excised tissue. In addition, PL profiles can be obtained efficiently by using ^31^P-NMR spectroscopy based on the organic phase of biphasic extracts, at no additional cost in terms of tissue material which is simultaneously extracted for water-soluble metabolites.

In addition to the techniques described above in Section 2.1, Section 2.2, Section 2.3, and Section 2.4, a variety of NMR spectroscopy methods for specialized applications will be presented in detail in Supplementary Materials. This concerns tissue extracts and suspensions, primarily in the domains of cancer and neurological research; however, various rare applications will also be discussed. Since studies of cancer metabolism are certainly the most prevalent applications of metabolic NMR spectroscopy of tissue extracts, protocols for the extraction of cultured cancer cells and NMR analysis of these extracts are extensively discussed in the literature [45]. Special attention has been given to the analysis of polar and non-polar lipids [53,54,55,56,57], sometimes in conjunction with molecular-biology-based analysis [58], and to the study of the incorporation of ^13^C-labeled metabolic precursors into the metabolism of cancer cells [40,41,59,60,61,62,63].

Moreover, NMR analysis proved to be useful in characterizing metabolic aspects of different forms of cell death, and of resistance to cell death [64,65,66,67,68,69,70,71,72]. In some of this work, “freezing” the metabolism of adherent cells before extraction was significantly accelerated by pouring (instead of pipetting) cell culture medium and cold rinsing liquid during the rinsing procedure [41,53,63,69]. Although nearly all tissue extract NMR studies in cancer research have been performed on experimental-animal tissues, rare examples of applications to human tissues also exist [73].

Instead of extracting metabolites from cultured cells and tissues for NMR analysis, it is possible and preferable to perform, for some NMR-active nuclei, metabolic ex vitro NMR measurements directly from static suspensions of harvested cells or excised tissue. This technique enables studies without tissue destruction and without magic-angle spinning. In the context of cancer research, this has been achieved for intact cancer cells [8,9,70,71,74], and for tissue samples from experimental animals [75,76]. Furthermore, numerous technical protocols of interest have been given by Euceda et al. [77].

Apart from oncology, neurology is the most prevalent domain of research that has benefited from results obtained by ex vivo metabolic NMR analysis. This is also true for studies of metabolic profiles of brain extracts from animal models, such as models of multiple sclerosis and mental retardation [78,79,80]. Moreover, the metabolome of neural and other stem cells has been investigated by tissue extracts and static cell suspension, in order to study the multidrug resistance of stem cells in general, and the metabolism of pluripotent embryonic stem cells in particular [8,53,81,82,83]. Furthermore, ex vivo metabolic NMR methods that fall within the scope of our review have been applied to a vast range of additional biological samples obtained from a variety of mammalian and non-mammalian species. Although reports on these applications are much sparser than publications on oncological or neurological applications, they will be briefly presented in Supplementary Materials (Section SI 1.4). These investigations concern studies of blood vessel tissue, skin, ascites cells, spermatozoa, serum lipids and milk lipids (mammalian origin) [84,85,86,87,88,89,90,91]; furthermore, measurements of mussels, corals, and plants (non-mammalian origin), as well as microorganisms (bacteria, yeast and other fungi, parasites) [92,93,94,95,96,97,98,99,100,101,102,103,104,105,106,107].

## 3. Discussion and Conclusions

### 3.1. Advantages of Metabolic NMR Analysis Based on Tissue Extracts vs. Intact Tissue

The review presented above and in Supplementary Materials highlights the fact that NMR spectroscopy of extracts of human, animal, and plant tissues, as well of cultured cells, continues to be a domain of research characterized by progress with respect to sample preparation and signal acquisition techniques. The strong emphasis on metabolomic studies has significantly contributed to the extension this method to new applications which are no longer limited to the, albeit still very much present, extracts of tissues from experimental animals and cancer cell extracts. On the other hand, recent developments such NMR spectroscopy by HR-MAS have begun to play a role at least as important as that of tissue extract NMR spectroscopy. Indeed, HR-MAS significantly increased the resolution of NMR spectra from intact (but excised) biological tissue when compared to spectra investigated without sample spinning. The HR-MAS method, just like the classical tissue suspension method, has the clear advantage that one crucial time-consuming step of the sample preparation process, metabolite extraction, is entirely avoided. However, particular physicochemical factors, as well as residual effects of tissue heterogeneity, prevent HR-MAS NMR spectroscopy of intact tissue from achieving the extremely high spectral resolution that can be obtained from optimized tissue extracts. Extracts can be processed such that major causes of line broadening are abrogated. One important cause of line broadening is the presence of certain ions that form complexes with metabolites to be studied. Such effects become particularly deleterious in ^31^P-NMR spectroscopy as phosphates easily form complexes with divalent ions. Here, line broadening is caused by intermediate rates of exchange processes between (mostly divalent) ions such as Mg^2+^ and phosphorylated metabolites such as nucleoside triphosphates (NTPs such as ATP, GTP, CTP, and UTP). As a consequence, the ^31^P-NMR resonances from these and other phosphorus nuclei are characterized by decreased signal-to-noise ratios and increased signal overlap, both factors significantly reducing the potential to detect and quantify individual metabolites. In addition, any presence of paramagnetic ions such as Fe^2+^, Fe^3+^, Mn^2+^, etc., may cause line broadening, to varying degrees for different nuclei. This effect is due to shortening of the spin-spin relaxation times (T_2_) of the observed nuclei; here, T_2_ is dominated by interactions of these nuclei with the strong magnetic moment of the unpaired electrons of paramagnetic ions. All of these effects can be virtually eliminated for extracts, either by masking otherwise complexing ions with the help of a chelating agent (EDTA, CDTA) added to an extract solution, or by removing these ions as a result of passing the extract solution through a cation exchange resin (Chelex) column before NMR spectroscopy.

Other advantages of extract (^1^H) NMR spectroscopy are the absence of (i) tissue water signal interfering with metabolite signals; (ii) the very broad signals from proteins and other macromolecules (usually visible in the form of gross baseline distortions); and (iii) broad lipid peaks interfering with water-soluble compounds of interest. In aqueous extracts, deuterated instead of protonated water is used as a solvent. Water-soluble proteins are dealt with in the extraction process through precipitation and subsequent removal by sample spinning. This is achieved either by first extracting the tissue of interest with perchloric acid (for aqueous extracts), or by using organic extraction solvents such as chloroform and methanol (for organic extracts). The latter two solvents are also used in so-called dual or biphasic extraction, as explained in the preceding sections of this overview. In this technique, water is added subsequently to methanol and chloroform, resulting in two separate extract phases: the aqueous phase (water-soluble metabolites in water/methanol) and the organic phase (lipids in chloroform/methanol). Since lipids are virtually insoluble in water, they are almost completely absent from aqueous extracts, and from the aqueous phase of dual extracts. A further advantage of extract NMR spectroscopy is the absence of residual line broadening due to microscopic gradients of magnetic susceptibility. The latter, a consequence of microscopic tissue structures such as cells, organelles, blood vessels, and others, are inevitable in intact tissue. Their effects on NMR line width can only be minimized by HR-MAS, but are altogether inexistent in metabolite solutions. Several protocols for tissue extract preparation and analysis by NMR spectroscopy are compared in Table S1 (Supplementary Materials).

### 3.2. Advantages of Metabolic NMR Analysis Based on Intact Tissue vs. Tissue Extracts

Since tissue or cultured-cell samples, investigated under HR-MAS or as static samples, are not extracted, there is no risk of introducing extraction artifacts into metabolite profile measurement. Metabolism of organisms predominantly occurs via biochemical reactions of small molecules dissolved in intracellular tissue water, these processes being catalyzed by enzymes. Metabolic in vivo NMR spectroscopy exclusively detects the “free” small molecules (metabolites) involved in these reactions. The reason for this is that any metabolites bound to proteins, other macromolecules or (semi)solid cell structures are undetectable (under conditions of slow or no exchange with the cytosol) because their short T_2_ results in extremely broad lines only contributing to deformations of the spectral baseline. By contrast, tissue extraction is known to liberate bound molecules (e.g., lactate) that are then detected in tissue extracts, together with free tissue metabolites. Thus, metabolite concentrations derived from extract NMR spectra may overestimate the amount of metabolite actually involved in relevant metabolic reactions. As a result, this artifact may lead to discrepancies with metabolic information obtainable through in vivo NMR spectroscopy. Of course, besides HR-MAS samples, also suspensions of excised tissue in buffer can serve to avoid this artifact. As indicated in the Introduction section, the latter approach is of particular interest in cases where no specialized equipment (HR-MAS probes) is desired or available for spectrum acquisition, as probes and NMR tubes commonly used in routine analytical NMR spectroscopy are entirely adequate. In fact, there is virtually no overlap between NMR spectroscopy studies employing static and HR-MAS tissue samples, as nearly all metabolic HR-MAS experiments published were based on ^1^H-NMR, while nearly all metabolic NMR spectroscopic investigations performed with static samples of excised tissue or cultured cells were based on heteronuclear NMR (mostly ^31^P, ^13^C and ^19^F). Classical tissue suspension NMR spectroscopy has the advantage that relatively large fractions of a given tissue (e.g., animal tumor or organ) can be fitted in the NMR tube (frequently 10 mm diameter tubes are used). For rat and, in particular, mouse experiments, this means that an entire tumor or organ (e.g., a minced kidney) can be fitted into the NMR tube, and a spectrum representative of the entire tissue in question can be acquired. By contrast, a sample as small as those used in HR-MAS (biopsy-sized) may not be representative of the entire tissue of interest. This is a “compensating advantage” to the general disadvantage that more biological material is needed for tissue suspension than for HR-MAS measurements.

### 3.3. Choice of Appropriate Protocols

Independently of the sample form and NMR nucleus used, the application of appropriate resolution enhancement techniques such as Lorentzian–Gaussian line shape transformation is very beneficial for identification and quantitation of NMR resonances if the parameters involved (i.e., line-broadening, LB, for Lorentzian deconvolution, and Gaussian broadening, GB, for Gaussian re-convolution) are optimized. The optimal values for these parameters depend on experimental conditions, i.e., sample type, measurement temperature, nucleus observed, magnetic-field strength, and so forth. Ultimately, the optimization of LB and GB (or analogous parameters for other resolution enhancement methods) has to be based on the characteristics of the actual raw spectra acquired, and is best done iteratively, i.e., by trial and error. We provide a practical example for such parameter values (legend to panel C of Figure 2). 

Several of the papers discussed in this overview also contained 2D NMR spectra for assignment of signals whose molecular origin was uncertain, even though we did not describe these explicitly in all cases. Moreover, several published papers have focused on signal assignment relevant to tissue metabolism, although some of this work has not been performed on tissues but on biofluids. Since peak assignment is not a major focus of our review, we will not discuss this topic in detail; the interested reader is invited to consult the references given here [71,106,107,108,109,110].

Overall, there are many techniques and protocols that can be applied, depending on the scientific information sought. In applications to metabolomics, oftentimes only semi-quantitative evaluations are of interest; here, statistical methods such as principal component analysis and discriminant analysis are directly applied to spectral portions (“buckets”) without referring to actual metabolite concentrations. However, for direct comparison with results from other biological analyses, full quantitation is needed to obtain meaningful information on biochemical processes. Thus, it is obvious that no one technique and, even more so, no one protocol can fit all applications. This means that there is an intrinsic limitation to attempts of introducing strict standardization of ex vivo and in vitro metabolic NMR analysis. Ultimately, it is the researcher in charge of a given project who should independently, judiciously, critically, and fully aware of the rationales of alternative approaches, assess existing methods and make use of these (with or without modifications) in a way that best suits the scientific aims of the research project in question.

## Data Availability

Not applicable.

## Supplementary Materials

The following supporting information can be downloaded at: https://www.mdpi.com/article/10.3390/molecules27134214/s1, Supplementary File S1: Supplementary Information. Reference [111] is cited in the Supplementary Materials.
