# Supplementary material for: Methodological Developments for Metabolic NMR Spectroscopy from Cultured Cells to Tissue Extracts: Achievements, Progress and Pitfalls
Source: Molecules. 2022 Jun 30;27(13):4214. doi: 10.3390/molecules27134214 (PMC9268249; doi:10.3390/molecules27134214)
Supplement: Supplementary file 1 [file molecules-27-04214-s001.zip › molecules-1723948-SI.pdf]

Supplementary Materials

Methodological Developments for Metabolic NMR Spectroscopy from Cultured Cells to Tissue Extracts: Achievements, Progress and Pitfalls

Norbert W. Lutz * and Monique Bernard

CRMBM, CNRS, Aix-Marseille University, 13005 Marseille, France; monique.bernard@univ-amu.fr

* Correspondence: nlutz@umars.fr

SI 1. Technical Details and Special Applications of Metabolic Ex Vivo NMR Spectroscopy

SI 1.1. Analysis of Tumor Metabolism

SI 1.1.1. Extracts of Cultured Cancer Cells, and of Tumor Tissue from Humans and Experimental Animals

Rinsing and Extraction Protocols for the Extraction of Cultured Cancer Cells

In Section 2.4.2. of the main body, a method for speeding extraction of cultured HeLa cells was presented [1]. The authors discussed the influence of different cell rinsing protocols on metabolite yields, and suggested the use of methanol/water/chloroform as extraction solvent [1]. Further details of their experiments are given in the following. In their tests, both the number of rinses (n = 1, 2 or 3) and the amount of PBS used per rinse (3 or 6 mL) was varied, each rinsing procedure taking up to several minutes. The authors found that the total amount of "metabolites" detected by ^{1}H-NMR spectroscopy in the PBS used for rinsing increased with both the number of rinsing steps, and the PBS volume used. However, there are no data indicating that this finding is indeed matched by an equivalent decrease in the content of the corresponding intracellular metabolites measured upon cell extraction. A separate experiment showed that intracellular glucose was markedly reduced already 30 to 60 s after cells had been transferred from high-glucose to glucose-depleted culture medium. The authors concluded that, therefore, leaking of metabolites into the rinsing liquid should be minimized by using no more than one rinse with 3 ml PBS, which should also minimize rinsing effects on glucose metabolism.

They also decided to recommend the simple protocol over the more time-consuming dual (or biphasic) extraction based on methanol/water/chloroform. However, their comparison of these two extraction methods seems to neglect the fact that the methanol/water/chloroform method is generally used to extract not only water-soluble metabolites but also lipids, whereas Mili et al. only evaluated the former. In addition, the methanol/water/chloroform extraction version used as reference method implies that the cellular compounds may undergo some metabolism before, during and after cell scraping because methanol alone does not completely denature proteins [1]. The authors did not report data based on shock-freezing the cells with liquid nitrogen right after a rinse with cold PBS, followed by scraping the cells into methanol at a temperature close to the melting point of methanol, upon which chloroform and water are added [2].

Phospholipid Analysis by NMR Spectroscopy of Cancer Cell Extracts

An example of the, albeit limited, potential of lipid analysis by ^{1}H-NMR spectroscopy is Mori et al.'s work in which they compared levels of selected (i) water-soluble PL metabolites (choline, PC and GPC) and (ii) lipids between cultured human breast and prostate cancer cells on the one hand, and solid tumor xenografts derived from these cells on the other [3]. While PC, GPC and choline were quantified from the aqueous extract phase, the organic phase enabled measuring ^{1}H-NMR signals from (predominantly) phosphatidylcholine ("PtdCho"), phosphatidylethanolamine ("PtdEt"), as

well as signals from (unspecified) saturated (–$(CH_2)_n$–) and unsaturated fatty acids (–CH=CH–). In addition, –CH_3 signals (saturation-unspecific) and signals from cholesterol (C_{18}) were detected. Although individual lipid subclasses or species could not be distinguished by this method (apart from cholesterol), the available spectral resolution was sufficient to determine that the degree of lipid unsaturation estimated from –CH=CH–/–CH_3 or –CH=CH–/–$(CH_2)_n$– ratios was significantly and consistently higher in tumors than in cultured cells. The ratios –N–$(CH_3)_3$/–CH_3 (assigned to PtdCho) and –C$\underline{H}_2$–$^+NH_3$/–CH_3 (assigned to PtdE) were also determined to estimate the relative prevalence of PtdCho and PtdE in cultured cells vs. tumors. These data were interpretable in conjunction with protein levels for enzymes crucial for lipid metabolism.

Note that each of the PL signals designated (i) "PtdCho" and (ii) "PtdE" in these ^{1}H-NMR spectra actually represents multiple PL subclasses, namely (i) the sum of PtdCho (=diacyl-PtdCho), alkyl-acyl-PtdCho, and choline-plasmalogen ($PtdCho_{plasm}$); and (ii) the sum of PtdE (=diacyl-PtdE), alkyl-acyl-PtdE and ethanolamine-plasmalogen ($PtdE_{plasm}$), respectively. Each of these PL (sub)classes, and many more, can be identified and quantified individually by ^{31}P-NMR spectroscopy of tissue extracts, provided the solvent has been optimized. A detailed description of the experimental conditions that are required for reproducible PL ^{31}P-NMR spectra has been published by Lutz et al. [4–6]. Although this method was originally based on the analysis on brain extracts (see Section SI 1.2.5 below), it can easily be adjusted to tumor and other tissues.

Combination of Metabolic NMR Spectroscopy of Cancer Cell Extracts with Molecular-biology-based Analysis

In many cancer lipid studies, NMR spectroscopy plays a supportive or auxiliary role rather than a main role. For instance, Gadiya et al. investigated interactive properties of two enzymes of the Kennedy pathway of choline-containing PLs, namely phospholipase D (PLD1, which converts PtdCho to phosphatidic acid (PA) and cho), and choline kinase-α (Chk-α, which phosphorylates free choline (cho) to PC), in cultured breast cancer cells and human breast cancer tissue [7]. They combined immunoblotting, a technique to determine enzyme expression at the protein level, with qRT-PCR, a technique to measure enzyme expression at the mRNA level, to analyze cultured cells in which PLD1 or Chk-α (or both) were suppressed by way of the siRNA method. Here, ^{1}H-NMR spectroscopy of the aqueous phase of cell extracts served to quantify PC, GPC, and cho to provide a link between the alterations in PLD1 and Chk-α activities caused by the corresponding siRNA treatments on the one hand, and the metabolic consequences on the concentrations of the water-soluble products or substrates of the reactions catalyzed by these enzymes, namely cho and PC, on the other. The evaluation also included the PC/GPC ratios found to be correlated with malignancy and metastatic potential in human cancers. Although only three peaks were evaluated in each spectrum, these experiments played a crucial role in elucidating the biochemical processes at the origin of features of cancer that are often observed by in vivo ^{1}H- and ^{31}P-NMR spectroscopy in the clinic and in experimental animals, namely, increased tot cho levels (=sum of cho, PC and GPC; ^{1}H-NMR) and PC/GPC ratios (^{31}P-NMR). Note that separate peaks

for PC and GPC can be obtained in high-quality, high-field (≥7.0 T) in vivo ^{31}P-NMR spectra but not in in vivo ^{1}H-NMR spectra.

Metabolic Pathway Analysis in Cancer Cells by 2D ^{1}H/^{13}C-NMR Spectroscopy of Extracts

An interesting study of extracts from cultured breast cancer cells and from mouse xenografts based on the same cell lines has recently been presented by Lane et al. [8]. The purpose of this work was to probe the metabolic phenotype of breast cancer cells. This was achieved by feeding cells and mice with isotopically enriched metabolic precursors, and by analyzing the metabolic fate of multiple isotope labels with NMR spectroscopy and MS in aqueous and organic tissue extracts (obtained with 10% trichloroacetic acid and 60% acetonitrile, respectively). The metabolic precursors comprised [U-^{13}C]-glucose, [^{13}C-1], [^{13}C-2]-glucose, [U-^{13}C,^{15}N]-glutamine, [U-^{13}C]-octanoate, and/or [U-^{13}C]-glycerol. This approach permitted the authors to not only follow particular biochemical pathways, but to obtain an overview over a vast metabolic network, in tune with their metabolomic objectives. For instance, their 2D TOCSY ^{1}H-NMR spectra revealed ^{13}C satellite cross-peaks for particular protons in the ribosyl units of adenine and uracil. Since these ^{13}C labeling patterns are consistent with the presence of $^{13}C_5$-ribose subunits in both types of nucleotides, an operation of the pentose phosphate pathway (PPP) via the oxidative and/or non-oxidative branches has been suggested. Other 2D TOCSY ^{1}H-NMR experiments with cells grown in [U-^{13}C]-glucose or [U-^{13}C,^{15}N]-glutamine demonstrated that the degree to which ^{13}C label is incorporated into the uracil ring at C-5 and/or C-6 from either of these precursors (Fig. 4, main body), depended on the breast cancer cell line used (MCF-7, MDAMB-231, or ZR75-1). Thus, the use of a relatively simple ^{1}H-NMR experiment enables the quantitation of isotopic enrichment of heteronuclear labels in biochemically relevant water-soluble metabolites, without the need to perform heteronuclear (^{13}C or ^{15}N) NMR spectroscopy.

Direct Quantitation of Specific ^{13}C Enrichment in Phosphorylated Metabolites of Cancer Cells by High-Resolution ^{31}P-NMR Spectroscopy of Extracts

In lipid extracts, NMR analysis of the type described in the preceding paragraph only provided information on the ^{13}C positional isotopomers of (phospho)lipids as a whole ("global lipid analysis" [9]), but could not distinguish the labeling patterns of individual PLs. This is why Lane et al. employed the high-resolution and accurate mass capability of FT-ICR-MS (Fourier Transform Ion cyclotron resonance mass spectrometry), which is able to obtain a large number of lipids and their ^{13}C isotopologue distribution directly from crude lipid extracts [10]. Nonetheless, ^{31}P-NMR spectroscopy has been shown long ago to allow the detection of a particular type of ^{13}C enrichment in PLs through the presence of ^{31}P-^{13}C J-coupling stemming from the choline moiety in the polar head group of PtdCho, based on ^{13}C-labelled choline as substrate [11]. Others have demonstrated that measurement of ^{13}C enrichment by ^{31}P-NMR is also feasible for a variety of water-soluble phosphorylated metabolites, through the presence of ^{31}P-^{13}C *J*-coupling visible in ^{31}P-NMR spectra of aqueous cell extracts [12,13]. For the colon carcinoma cell line, HT-29, these metabolites included several [1-^{13}C]-glucose-derived

triose and hexose phosphates, GPE, GPC, nucleotides (ATP, GTP), and five different UDP-hexoses [12]. The ^{31}P-^{13}C coupling constants ranged from 5 to 7 Hz, as confirmed by ^{13}C-NMR spectroscopy for the peaks identifiable in less crowded spectral regions. Specific enrichments were compared between HIV-1-infected HT-29 cells and uninfected control cells. Although ^{13}C enrichment in lactate was significantly increased upon HIV-1-infection (as determined by ^{1}H-NMR spectroscopy), no significant change in ^{13}C enrichment was detected for the phosphorylated compounds mentioned above [13]. These cells were extracted using perchloric acid, similar to the procedure described in Section 2.4.1 (main body). After rinsing with ice-cold standard saline, liquid nitrogen was poured directly into the flasks. When the monolayer was frozen 0.9 mol/l $HClO_4$ solution was added. Cells were scraped from the flask surface with a rubber policeman, left standing on ice for 15 min, and the resulting mixture of cell debris and metabolite solution was centrifuged. Subsequently, the supernatant was neutralized with KOH, and the extract solution was lyophilized. The lyophilizate was redissolved in 1 ml D_2O. For high-resolution ^{31}P-NMR spectra, EDTA was added to complex the divalent ions in the solution, thus preventing complex formation between the phosphate moiety of the phosphorylated metabolites and divalent metals. The latter point is particularly critical to "mask" paramagnetic ions that could otherwise decrease ^{31}P T_2 values, and thus, render ^{31}P-NMR lines very broad, resulting in low spectral resolution. Finally, pH was adjusted to a value of about 6.65 [14]. Preceding experiments had shown that significantly higher pH values broaden ^{31}P-NMR resonances and thus prevent high-resolution ^{31}P-NMR analysis, whereas significantly lower pH values diminish chemical-shift differences between individual metabolites, notably for peaks in the PME (or sugar phosphate) region of the spectrum. The spectra achievable in this way are shown in Figure SI 1 for ^{1}H- and ^{31}P-NMR.

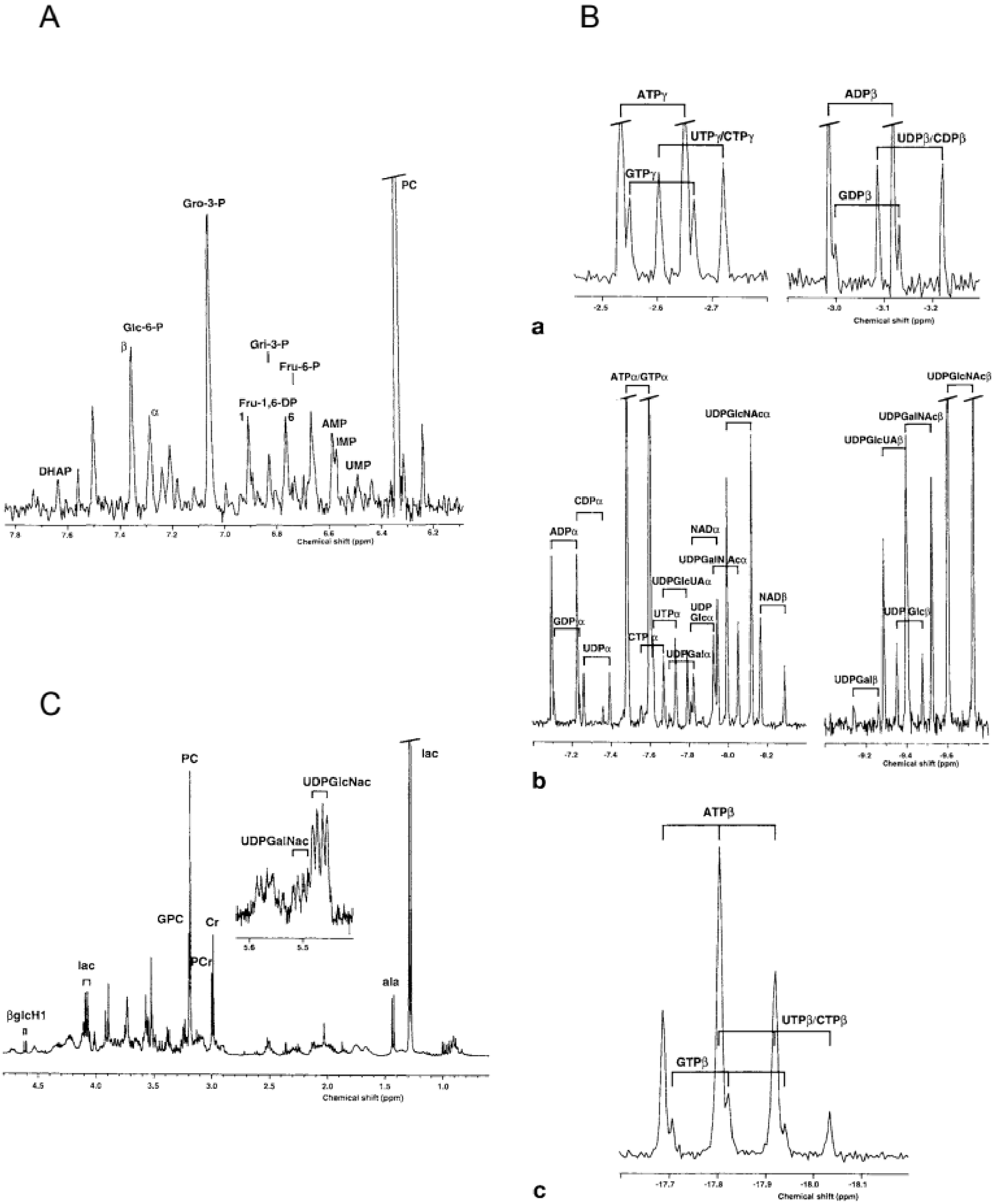

Figure S1. Panel A. PME region of a typical high resolution ^{31}P-NMR spectrum (9.4 T) from a HT-29 cell extract. The spectrum was acquired at 4 °C, pH was 6.65 ± 0.02. DHAP: dihydroxyacetonephosphate; for other abbreviations see text. Panel B.

Nucleotide regions of a ^{31}P-NMR spectrum (9.4 T) from a typical HT-29 cell extract. The spectra shown have been individually processed for optimum resolution rather than for optimum quantitation. The individual regions are plotted with different intensity scalings. Panel C. A typical ^{1}H-NMR spectrum (9.4 T) from a PCA extract of HT-29 cells. Reprinted with permission from [14].

Extracts of Radio-Resistant Cells

Shen et al. studied metabolite profiles related to radio-resistance for two malignant non-small cell lung carcinoma (NSCLC) cell lines, CL1-5 and CL1-0 [15]. As CL1-5 was more resistant to radiation than was the parental CL1-0 cell line, these cell lines were considered a suitable model to investigate changes in metabolite profiles related to radiation exposure. After two washes with ice-cold PBS solution, cells were placed on ice and "suspended" in 1 mL ice-cold MeOH and deionized water (1:1, *v*/*v*) for 10 min. However, it is uncertain whether the authors assumed (or verified) that the exposure to this solvent mixture resulted in a release of all water-soluble intracellular metabolites into the solvent. Their protocol continues stating that the extract was centrifuged at 1,800 × *g* at 4 °C for 15 min, and that the supernatant was collected and stored at −80 °C until use. The cell extract was thawed at room temperature and evaporated to dryness using nitrogen gas. The dried sample was redissolved in 200 μL D_2O containing 1 mg/mL of 3-trimethylsilyl-(2,2′,3,3′-$^{2}H_4$)-1-propionate (TMSP) for "intensity" quantitation by ^{1}H-NMR spectroscopy (no absolute concentrations were provided). The authors evaluated the spectral region between 0.2 to 4.4 ppm by binning (binning size 0.01 ppm). The relative signal intensities of these bins were directly subjected to PCA (Principal Component Analysis). The PCA score plots demonstrated clustering for the CL1-0 and CL1-5 cells as a function of time after radiation treatment; clustering was most unambiguous for the longest delay investigated (24 h). The PCA loadings indicated peak regions with statistically significant differences between the two cell types, corresponding to metabolites of interest. According to the authors, the primary metabolite that determined the radio-sensitivity was glutathione, which could protect cells against oxidative stress as it has antioxidant properties. The higher endogenous levels in CL1-0 cells of metabolites such as glutathione, creatine phosphate, glutamate, PC, pyroglutamate, and taurine were hypothesized to be key to the protection against radiation induced damage. This investigation illustrated a fundamental feature of metabolomic studies, i.e., their hypothesis-generating character, while mechanistic questions can rarely be answered by metabolomics.

Extracts of Cells Resistant to Apoptosis and Oxidative Stress

Resistance to oxidative stress also was the topic of an entire group of publications dealing with metabolic effects of apoptosis induction and resistance [16–20]. Mouse thymic lymphoma tissue culture cells, WEHI7.2, were subjected to dexamethasone (DEX) treatment, and the metabolic response to this treatment was analyzed by ^{1}H- and ^{31}P-NMR spectroscopy of cell extracts for different time points after treatment. The purpose of this metabolomic study was to compare the WEHI7.2 wild-type and four WEHI7.2 variants which had been rendered resistant to oxidative stress and apoptosis by different modifications. In addition, the metabolic profiles of untreated

WEHI7.2 cells and its variants have been measured. Since WEHI7.2 cells grow in suspension rather than as adherent cells, they were harvested by centrifugation, then washed and fixed by adding methanol. Chloroform and water were added to the sample, and the phases were separated by incubation at −20 °C overnight and subsequent centrifugation as described above for methanol/chloroform/water extracts of adherent cells. After solvent removal, the lipid sample was dissolved in a ^{2}H-chloroform–methanol–water solution containing EDTA to mask divalent ions. The lyophilizate of the aqueous phase was dissolved in a D_2O solution also containing EDTA. While ^{1}H- and ^{31}P-NMR spectra were acquired to quantitatively analyze the water-soluble metabolites, ^{31}P-NMR spectroscopy was used to analyze the concentrations of a wide range of PL classes and subclasses. Although the four WEHI7.2 variants had acquired resistance to oxidative stress and apoptosis through very different mechanisms of action, they exhibited metabolic similarities that enabled a general characterization of metabolic properties contributing to resistance. The most important characteristic was the metabolic flexibility of the DEX-resistant vs. the DEX-sensitive cells, observed across several metabolic pathways. This is also the first metabolic NMR work to analyze glutaminolysis and its role in resistance to oxidative stress and apoptosis [19].

Another work dealing with metabolic effects of apoptosis induction was based on several combinations of TNF-α and IFN-γ treatments over 4 to 24 h [21]. Adherent cells (colon adenocarcinoma, HT-29) were extracted using the perchloric-acid extract, and treatment effects on energy, glucose and PL metabolism were detected by ^{1}H- and ^{31}P-NMR spectra. Since cells undergoing apoptosis detach from the bottom of the cell culture flask over time, and since only attached cells were extracted for NMR analysis, the metabolic results obtained characterized metabolism at an early stage of apoptosis induction. Because a simultaneous lactate and NTP increase only occurred in cells treated with both drugs, and because only these cells exhibited histologically confirmed drug-induced apoptosis, it was hypothesized that simultaneous lactate and NTP changes may serve as early markers of treatment response upon apoptosis induction, at least for some tumors.

Extracts of Human Tissues

As is obvious from the above, most NMR spectroscopic tissue extract studies to be found in the published literature have been based on cell culture or excised tissue samples from animal models. By contrast, most NMR investigations of human biopsies have not been performed on tissue extracts, but using the HR-MAS technique not discussed in detail here. One of the few papers on NMR spectroscopy of human tissue extracts has been presented by Guo et al. [22]. The authors intended to determine if in lung cancer, relative levels of choline and lactate are correlated with ^{18}F-FDG (^{18}F-fluorodeoxyglucose) uptake detected in vivo PET (positron emission tomography) before tissue excision, the latter parameter being negatively correlated with patient survival. This hypothesis was based on preceding findings in the literature that in some tumors, increased “choline” and lactate (Warburg effect) was associated with malignancy. However, the authors did not find a significant correlation. Even so, it should be noted that there are some missing details in Guo et al.’s work. First, the authors used the aqueous

phase of a water/methanol/chloroform extract to measure ^{1}H-NMR spectra. As shown in Section 2.4.2 (main body) for Haukaas et al., also Guo et al. reported that tissue specimens obtained from patients were stored at −20 °C "immediately" after dissection. Thus, the same question as to the meaning of "immediately" arises here. Metabolic changes, in particular glycolysis, can change drastically within seconds of ischemia induction by dissection; if this occurred in Guo et al.'s lung samples, the lactate levels detected might reflect lactate produced due to surgically caused hypoxia (in all samples), i.e., an artifact rather than the true lactate level in lung tissue immediately before resection. In this case, the lack of correlation between measured lactate levels with ^{18}F-FDG PET results and patient outcome would not be surprising. Moreover, the authors assigned one particular peak in their ^{1}H-NMR spectra to "choline", but it is unclear if this peak may overlap with other peaks of similar chemical shift. For instance, nearby peaks could be taurine, PC or GPC, but none of these labels is seen in Guo et al.'s spectra. If the signal in question is correctly assigned to choline, then, again, the lack of correlation between the level of this compound and ^{18}F-FDG uptake in PET would not be surprising; the in vivo methyl ^{1}H-NMR peak previously found to be correlated with malignancy was not choline, but tot cho (which is the sum of choline + PC + GPC). It has been widely shown by in vivo ^{31}P-NMR spectroscopy that in many malignant tumors, it is PC levels that are elevated, but Guo et al. did not refer to (or exclude) the possibility that PC may have been detected in their spectra. In summary, the way this study was performed was not conducive to conclusive results.

SI 1.1.2. Suspended Intact Cancer Cells

While the metabolic studies described in the preceding paragraphs have been carried out based on cell extracts, cells routinely cultured in suspension lend themselves quite naturally well to analysis by in vitro NMR spectroscopy of concentrated cell suspensions. Apoptosis induced by photodynamic therapy (PDT) is an interesting example showing that even for intact cells, the spectral resolution of ^{31}P-NMR spectra is sufficient to quantitate a number of individual energy, glucose and PL metabolites, at least for 1 h measurements [23] (see also Section SI.1.2.2 below, discussion of Ramm Sander et al.). The most original discovery of this work is the detection of a particular PL metabolite, CDP-choline, in cells undergoing apoptosis, but not in cells that have only been chemically sensitized to apoptosis, without being subjected to light irradiation for actual apoptosis induction. However, also the sensitized non-apoptotic cells suffered from significant metabolic perturbations when compared with sham-treated control cells, namely reductions in phosphocreatine (PCr), UDP-hexose and PDE levels (as percentage of total phosphate), and reduced intracellular pH. These and other metabolic studies of apoptosis effects in cultured cells by NMR spectroscopy have been reviewed in detail elsewhere [24,25].

Adherent cells have to be detached from the surface they grow on, and from each other (usually by trypsinization) before they can be placed in buffer to form a suspension that can be subjected to NMR spectroscopy (see also Sections 2.1.1 and 2.1.2, main body). This system has been used to study the influence of cell culture medium formulation on PME and UDP-hexose levels in colon carcinoma cells by proton-decoupled ^{31}P-NMR spectroscopy at 11.7 T

[26]. Here, cells were cultured in different media supplemented with different serum types, but also glucose and inositol concentrations varied between the media formulations used. The largest variation in intracellular metabolite levels was seen for PC and UDP-hexose (up to seven-fold changes). Remarkably, although the spectra were acquired from intact cells and not from extracts, the resolution obtained was sufficient to unambiguously discern, assign and quantitate up to three individual UDP-hexose species when using appropriate data processing (Lorentzian–Gaussian line shape transformation). For each of these, a separate ^{31}P-^{31}P ^{2}J doublet was seen. The methodological message of this work and similar works [27,28] is two-fold: (i) spectral resolution can be substantially improved even for intact cells if both experimental setup and spectrum processing are optimized; and (ii) to obtain reproducible spectra from cultured cells in general, it is imperative to use virtually identical culture media.

SI 1.1.3. Suspended Tissue Obtained from Experimental Animals

It has become clear from the preceding paragraphs that the vast majority of recent in vitro tumor NMR spectroscopic studies has been centered on ^{1}H-, whereas ^{31}P- and ^{13}C-NMR (with partial exception of hyperpolarized ^{13}C) have been much less common. Even rarer are in vitro ^{19}F-NMR studies. The latter are of interest when the metabolism of certain fluorinated drugs is to be followed. This group of investigations is mainly geared towards the metabolism of fluorinated anticancer pyrimidines, such as 5-fluorouracil (5-FU or FU), which can easily be followed by studies of excised tissues. In addition, the intended target of this drug (i.e., tumor tissue), organs such as liver, kidney, brain etc. of experimental animals can also be analyzed by subjecting these to ^{19}F-NMR. While rather concentrated fluorinated FU metabolites can be detected in vivo, less concentrated compounds require a more sensitive ex vivo technique. A major project of this type has been presented several years ago [29,30]. The purpose of this study was to investigate differences in FU metabolism underlying different drug delivery protocols [30]. Rats with DS sarcoma transplanted in the thigh were given FU in different doses multiple times, as systemic intravenous or locoregional (directly-to-the-tumor) intra-arterial treatment, over different time periods (from bolus injections to 24 h infusions). At the end of each treatment, rats were sacrificed and tumor, liver, and kidneys were rapidly excised, frozen in liquid nitrogen and stored at 25 °C until use. Each frozen tissue sample was cut into several small pieces which were placed in a chilled 10 mM diameter NMR tube and quickly weighed. Ice-cold HEPES saline buffer (pH 7.4) was added and transferred to the NMR spectrometer after stirring of tissue and buffer to remove air bubbles. Measurement at 4 °C was possible during approximately 1 h without significant metabolite loss (primarily fluorinated nucleotides). In addition, FU and its anabolites (several fluorinated nucleosides and nucleotides), also fluorinated catabolites (FU degradation products) were quantified, such as α-fluoro-β-alanine (FBAL), α-fluoro-β-ureidopropionic acid (FUPA), *N*-carboxy-FBAL (CFBAL), and fluoride. For the same animals, tumor volume vs. time during treatment, various blood parameters and survival times were recorded. This integrated multi-parameter study yielded a complex data set revealing interrelations between FU anabolism and catabolism, tumor regression and FU toxicity (deleterious

effects on vital organs, even affecting survival). The overall results suggested that low-dose (25 mg/kg) locoregional infusions over 5 or 24 h resulted in most efficient FU anabolism in the tumor, at maximum tumor regression and animal survival, avoiding marked FU anabolism in and toxicity effects on vital organs. Although tissue extraction would have resulted in even better spectral resolution (in particular for individual F-nucleoside and F-nucleotide resonances), the quality of spectra achieved for excised tissues was entirely adequate for the stated purpose of distinguishing FU as well as its anabolites and catabolites in the tumor and in different organs. In fact, the assignment of individual F-nucleoside and F-nucleotide peaks in the less resolved tissue spectra were based on aqueous test solutions of FU anabolites (prepared from original compounds) involved in fluoropyrimidine chemotherapy [29]. The results presented in this ^{19}F-NMR paper, that paid particular attention to the pH dependence of the peaks in question, were used to assign the corresponding F-nucleoside and F-nucleotide peaks in tissue spectra [30] (Figure S2). Further examples of metabolic NMR spectroscopy of cell suspensions are given in Section SI 1.2 below.

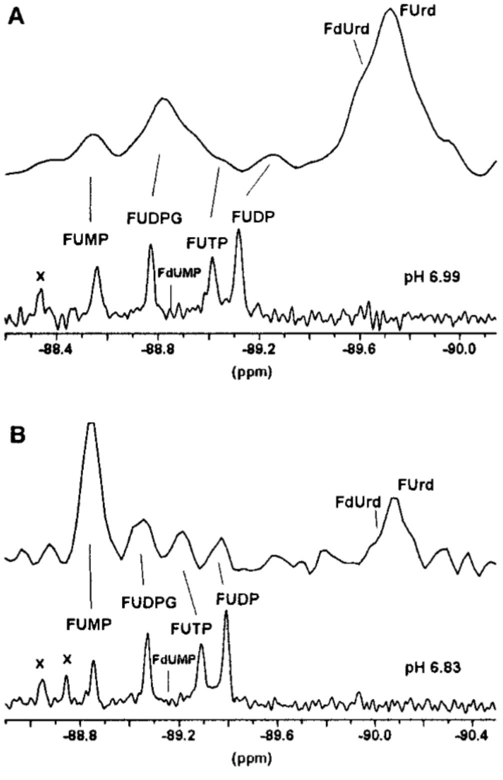

Figure S2. ^{19}F-NMR spectra (11.7 T, 4 °C, 1 h measurement) of intact tumor tissue (DS sarcoma, rat), excised following treatment with Fura (5-fluorouracil, 5-FU) (upper spectrum of each pair; (**A**) 100 mg/kg, 5 h infusion, locoregional i.a.; (**B**) 100 mg/kg, i.v. bolus, 1 h delay), are compared with spectra of model solution C (lower spectrum of each pair; (**A**) pH 6.99; (**B**) pH 6.83). The chemical composition of solution C is described in the reference given below. From the available reference spectra, those giving the best match for chemical shifts in the tissue spectra were chosen (e.g., alignment of FUMP signal). The expected position of FdUMP (not present in solution C) is marked in the reference spectra; the expected position of FdUrd is marked in the tissue spectra. The peaks marked with (x) are impurities (side products) in the

synthetic FUMP used. Quantitative analysis of the tissue spectra gave: (**A**) total Fnuctd (fluoronucleotides) = 89, F-Nucsd (fluoronucleosides) = 206; (**B**) F-Nuctd = 218, F-Nucsd = 90 (in nmol/g). Reprinted with permission from [29].

SI 1.2. NMR Spectroscopy of Stem Cells and Brain

SI 1.2.1. Neural Stem Cells

Loewenbrück et al. used ^{1}H-NMR spectroscopy of cell suspensions at 35 °C to determine whether a particular signal at 1.28 ppm is characteristic of neural stem cells (NSC) [31], as hypothesized previously in a *Science* paper [32]. A well-known, relatively broad resonance with this chemical shift has long been assigned to the methylene protons of mobile lipids that may accumulate in many cells under certain circumstances, e.g., after induction of apoptosis and/or necrosis. This lipid peak was thought to be of particular interest because it can be detected in vivo, and, therefore, could serve as a marker of neurogenesis in patients. To determine the specificity of the 1.28 ppm resonance for NSC, Loewenbrück et al. compared spectra of this cell type with those obtained from a wide set of undifferentiated and maturely differentiated cell populations, with or without apoptosis or necrosis induction. Neurospheres containing both glial and neuronal cells were studied in addition to pure astrocytic cultures, embryonic stem (ES) cells, ES-cell derived NSCs, induced pluripotent stem (iPS) cell-derived NSCs, and embryonic fibroblasts. Furthermore, melanoma and glioma cell lines were used. All cells were of murine origin. The authors found a correlation of areas under the 1.28 ppm peak, relative to the reference peak derived from a known amount of added trimethylsilylproprionate (TSP), with apoptosis, but not with necrosis, cell cycle phase distribution, cell size, or cell type. Although this peak was not specific for NSC per se, the authors suggested that its specificity for apoptosis as a major selection process during neurogenesis may potentially render this resonance still useful as an indirect marker of neurogenesis in vivo.

SI 1.2.2. The Stem Cell Metabolome

Following this work, Ramm Sander et al. published a review paper dealing with the specificity of NMR spectra for the stem cell metabolome in general [33]. They also arrived at the conclusion that the ^{1}H-NMR-visible mobile lipids reflect proliferative stress in general and apoptosis in particular, but are not useful as markers of cell types. It was pointed out that the usefulness of NMR spectroscopy in analyzing metabolic profiles generally depends on the technique used, and that the interpretation of the results has to take into account the experimental conditions (in vivo vs. in vitro; intact cells vs. cell extracts; differentiated vs. undifferentiated cells). For instance, in fast growing cells (especially in tumor cells), increased tot cho is one of the major conspicuous findings both in vitro and in vivo, but in vivo ^{1}H-NMR spectroscopy is unable to discern between the three possible contributions to the tot cho methyl resonance, representing PC, GPC and choline. Here, the statement by Ramm Sander et al. that it is "not yet possible in NMRS of intact cells …[to resolve] the different species of choline-containing compounds phosphocholine [and] glycerophosphocholine" is clearly inaccurate because the required resolution is available through ^{31}P-NMR spectroscopy of intact

cells, both in suspension and embedded in a gel during perfusion [23,26–28,34].

SI 1.2.3. Multidrug Resistance of Stem Cells

While Section 1.1.1. included several examples of metabolic NMR analysis of resistance to treatment in cancer cells in general, one particular form of drug resistance appears to be closely linked to a cellular mechanism that is able to export a variety of drug molecules that have already entered the cells. This phenomenon is known as multidrug resistance (MDR) and is often based on transporters such as the P-170 glycoprotein, acting as a "pump" for these molecules. ^{31}P-NMR spectroscopy of cell suspensions (measured at 4 °C), and metabolically active, perfused cells (measured at 37 °C) has been used to assess metabolic differences between two renal cell carcinoma (RCC) cell lines, KTCTL-26 and KTCTL-2, with high and low expression of P-170 glycoprotein, respectively [28]. Differences in response to drug treatment were investigated for incubations with various doses of vinblastine (VBL) alone or as cotreatments with various concentrations of the calcium antagonist diltiazem (DIL) for resistance reversal, and/or with interferon−α (IFN-α) which is frequently used in combination with VBL in the clinic. KTCTL-2 and KTCTL-26 cells exhibited significant inherent differences in PC, GPC, GPE, and PCr levels. It was concluded that metabolomic ^{31}P-MRS detects different metabolite profiles for RCC cell lines with different MDR phenotypes and may be useful for characterization of tumors in a clinical setting. However, since KTCTL-2, despite its low P-170 expression, exhibited a higher resistance to VBL/DIL treatments than KTCTL-26, other mechanisms of drug resistance must have been operative in this case, e.g., resistance based on other members of the ATP-binding cassette (ABC) transporter family [28].

One particular ABC transporter, ABCB5, has been shown to be characteristic of cancer stem cells, and two groups of human G3361 melanoma cells, the wild-type with intact ABCB5 expression (ABCB5-WT) and a corresponding variant with inhibited ABCB5 expression (ABCB5-KD) were compared by metabolomic analysis of methanol/chloroform/water extracts [2]. Both water-soluble metabolite (^{1}H- and ^{31}P-NMR) and PL (^{31}P-NMR) profiles were studied. Absolute and relative metabolite levels yielded significant differences for compounds involved in glucose, amino acid, and PL metabolism. By contrast, energy metabolism was virtually unaffected by ABCB5 expression. Redistributions within the PL pool suggested enhanced membrane PL turnover as a consequence of ABCB5 expression. These metabolomic results suggested that the underlying biochemical pathways may offer targets for melanoma therapy, potentially in combination with other treatment forms [2].

SI 1.2.4. Pluripotent Embryonic Stem Cells

Ramm Sander et al. noticed that on the one hand, in vivo ^{1}H-NMR spectroscopy had shown that in early postnatal rodent brains tot cho decreased with maturation, as is the case for the human fetal brain during gestation [33]. On the other hand, pluripotent ESCs in vitro were also characterized by marked tot cho signals when compared with neuralized embryonic stem cells (NSCs) and other differentiated neural cell types, for example, neurons, astrocytes, and even compared with various tumor cell

lines. In these ESC studies, high-resolution ^{1}H-NMR of cell extracts was used to discriminate methyl signals of free choline, PC and GPC. Surprisingly, GPC was found to be absent in pluripotent ESCs, whereas PC was most abundant. Upon differentiation, however, the ratio of PC to GPC decreased in both ESCs and CSCs. These results underline the importance of experimental conditions (including in vivo vs. in vitro and growth media), differentiation status, stemness, measured nucleus (^{1}H vs. ^{31}P) and other factors for the metabolic profiles obtainable from the cells under consideration.

SI 1.2.5. Metabolic Analysis of Brain: Animal Models of Multiple Sclerosis and Mental Retardation

Experimental autoimmune encephalomyelitis (EAE), an established rat model of multiple sclerosis, was used to investigate EAE effects on metabolic profiles of brains. These effects were compared with effects of adjuvant arthritis (AA) [35]. Interestingly, both inflammatory diseases caused similar changes in metabolites involved in regulation of brain cell size and membrane production: among the osmolytes, taurine and the neuronal marker, *N*-acetylaspartate (NAA), were decreased, and the astrocyte marker, *myo*-inositol, was slightly increased in the EAE and AA groups compared with controls. In addition, ethanolamine-containing PLs, sources of inflammatory agents, were increased in both groups, while aspartate and isoleucine were less concentrated for EAE than for AA. The protocol for sample preparation was virtually identical for both studies discussed in this section: rapidly freeze, in liquid nitrogen, the brains immediately after removal from the skulls; store at −80 °C until extraction; cut frozen brain, under liquid nitrogen, into smaller pieces for weighing; and quickly homogenize brain pieces in cold methanol before adding chloroform and water. The protocol had been specifically elaborated for brain extracts [4,5]. While extraction per se was straightforward, particular attention was given to five parameters involved in the preparation of the final PL solution to be subjected to ^{31}P-NMR spectroscopy: volume proportions of methanol vs. chloroform vs. water; amount of chelating agent in, and pH of the aqueous component of the solvent; amount of solvent per gram tissue extracted; and measurement temperature. With all of these parameters optimized, excellent reproducibility of results was obtained, as documented in the validation papers for this method [4,5]. These papers also specify optimized NMR acquisition and processing parameters needed for best PL quantitation. As indicated in Section SI 1.1.1, this protocol can be easily adjusted to extracts of tissues other than brain.

Metabolomic ^{1}H- and ^{31}P-NMR spectroscopy of methanol/chloroform/water extracts of brain tissue has been used to establish metabolic profiles characteristic of neurological diseases. For instance, a mouse model of Rett syndrome, a form of mental retardation mostly caused by mutations in the MECP2 gene, has been studied by comparing control mice with Mecp2-deficient mice [36]. This in vitro study was a follow-up on in vivo work for the same animal model [37], with the aim to analyze substantially more metabolites than was possible by way of the in vivo approach. Although HR-MAS NMR analysis of brain tissue is now rather well established, extract studies have the benefit that the metabolite solutions used for analysis are extremely stable since enzymes have been denatured and removed from

metabolic substrates. This avoids metabolic sample degradation over long measurement periods. In addition, lipid and macromolecule signals, responsible for baseline distortions, do not hamper quantification of water-soluble metabolites, and the spectral resolution is increased compared with HR-MAS spectra. Moreover, PL profiles, being of particular interest since brain tissue contains large amounts of myelin, can be analyzed when in solution, but not based on the semi-solid HR-MAS samples. This study established, for the first time, detailed metabolic fingerprints of perturbed brain growth, osmoregulation, and neurotransmission in a mouse model of Rett syndrome. Decreased levels of the astrocyte marker, *myo*-inositol, as well as reduced choline phospholipid turnover were detected in *Mecp2*-deficient vs. wild-type mice. In addition, PL profiles revealed alterations of the platelet activating factor (PAF) cycle in *Mecp2*-deficient animals.

SI 1.3. Studies of The Metabolism of the Extracellular Space

The metabolite profiles discussed in the preceding sections represent exclusively (or overwhelmingly, when excised tissues are studied) intracellular metabolites. By contrast, Kostidis et al. presented a very complex, detailed protocol for separately recovering, extracting and analyzing, by ^{1}H-NMR spectroscopy, intracellular and extracellular metabolites from cultures of adherent and suspension cells [38]. The use of a high-field NMR spectrometer (14.1 T) in conjunction with 3 mM NMR tubes enabled them to obtain highly resolved 1D and 2D spectra (Figure S3). In addition to the usually reported metabolites, they also showed that a number of phosphorylated metabolites can, under these conditions, be identified by spiking, although the ^{1}H-NMR resonances of these metabolites (in particular, UDP-hexoses, UDP-hexosamines and certain sugar phosphates) heavily overlap with other resonances (see also Figure S1), not unlike in previously reported ^{1}H-NMR spectra obtained at 9.4 T [14]. For this reason, accurate quantitation of a wide range of phosphorylated glucose metabolites and nucleotides is better performed by ^{31}P-NMR spectroscopy of extracts [14]. Of course, in this case cells must not be rinsed with phosphate buffer but with a different (e.g., bicarbonate) buffer that does not interfere with ^{31}P-NMR. Although the authors suggested a variety of extraction solvent mixtures with different polarities, they (as nearly all other authors discussed in this overview) did not consider solvent mixtures that would enable collection of both water-soluble metabolites and lipids. However, the latter have been described elsewhere, supplemented with an educational video [39].

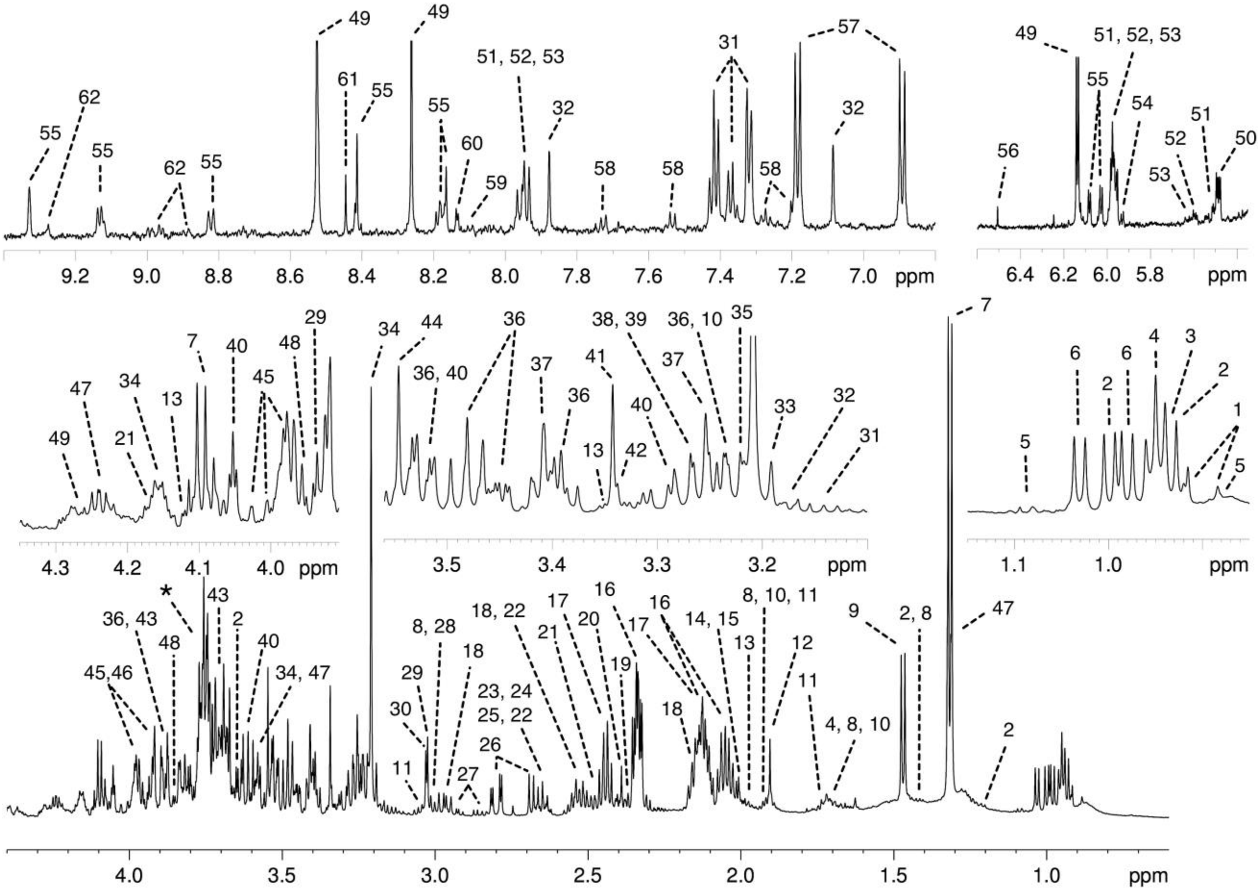

Figure S3. Regions of 600 MHz ^{1}H-NMR spectra (14.1 T) of BHP2-7 cells extract dissolved in deuterated phosphate buffer. Peak assignments: 1: pantothenate; 2: isoleucine; 3: 2-oxoisocaproate; 4: leucine; 5: 3-methyl-2-oxovalerate; 6: valine; 7: lactate; 8: lysine; 9: alanine; 10: arginine; 11: ornithine; 12: acetate; 13: proline; 14: *N*-acetylaspartate; 15: *N*-acetylglutamine; 16: glutamate; 17: glutamine; 18: glutathione (GSH); 19: pyruvate (oxalacetate); 20: succinate; 21: pyroglutamate; 22: citrate; 23: methionine; 24: hypotaurine; 25: malate; 26: aspartate; 27: asparagine; 28: α-ketoglutarate; 29: creatine; 30: phosphocreatine; 31: phenylalanine; 32: histidine; 33: choline; 34: *O*-phosphocholine; 35: *sn*-glycerophosphocholine; 36: D-glucose; 37: taurine; 38: betaine; 39: trimethylamine-*N*-oxide; 40: *myo*-inositol; 41: methanol (residual solvent); 42: hypotaurine; 43: galactitol; 44: glycine; 45: fructose; 46: galactose; 47: threonine; 48: serine; 49: ATP; 50: galactose-1-phosphate; 51: UDP-*N*-acetylglucosamine; 52: UDP-glucose; 53: UDP-glucuronate; 54: UMP; 55: NAD.; 56: fumarate; 57: tyrosine; 58: tryptophan; 59: GTP; 60: nicotinate adenine dinucleotide; 61: formate; 62: 1-methylnicotinamide; * overlapped peaks from D-glucose, D-galactose, Fructose, GSH and several amino acids (–CaH–). Reprinted under the Creative Commons Attribution License (CCAL) terms from [38].

As for water-soluble extracts, Kostidis et al. payed much attention to effectively remove proteins; the latter point was discussed in detail by Álvarez-Guerra et al. for blood extractions [40]. Gómez-Archila suggested optimized methods for isolating and processing peripheral blood mononuclear cells (PBMC) for ^{1}H-NMR-based metabolomic profiling, resulting in identification of a particularly vast range of metabolites (Figure

S4) [41]. Since these protocols did not contain major aspects not yet covered by other work discussed here, we will not discuss these in detail. Moreover, although biofluids of mammals and experimental animals constitute "extracellular space", these fluids will not be dealt with in this review focused on tissue.

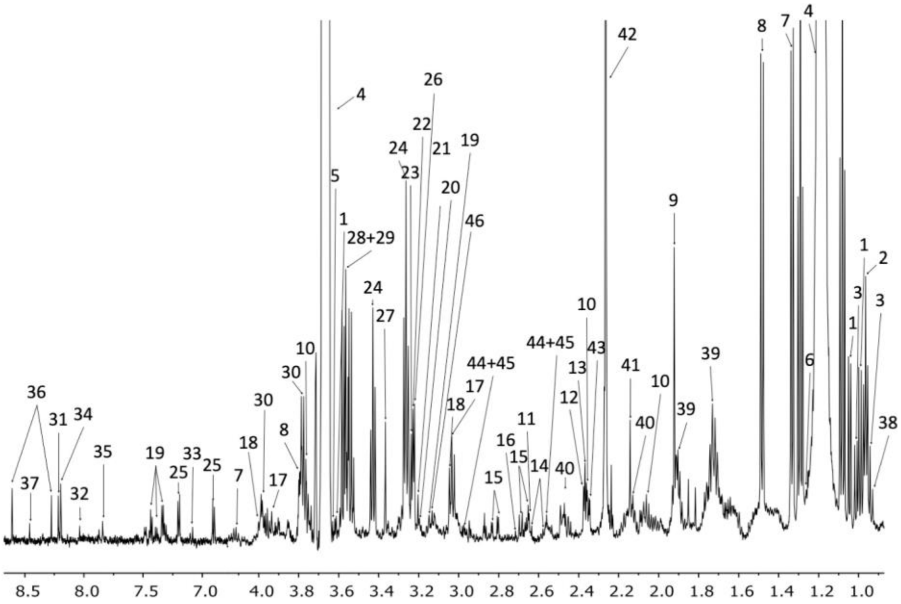

Figure S4. The assigned ^{1}H-NMR spectrum (14.1 T) of peripheral blood mononuclear cells (PBMC) isolated and processed using an ultrasound and ultrafiltration method given in the reference below. 1: valine, 2: leucine, 3: isoleucine, 4: ethanol, 5: threonine, 6: 3-hydroxyisovalerate, 7: lactate, 8: alanine, 9: acetate, 10: glutamate, 11: methionine, 12: pyruvate, 13: succinate, 14: citrate, 15: aspartate, 16: sarcosine, 17: creatine, 18: creatinine, 19: phenylalanine, 20: choline, 21: *O*-phosphocholine, 22: carnitine, 23: betaine, 24: taurine, 25: tyrosine, 26: trimethylamine *N*-oxide, 27: methanol, 28: glycine, 29: glycerol, 30: serine, 31: inosine, 32: GTP, 33: xanthurenate, 34: oxypurinol, 35: xanthine, 36: AMP, 37: formate, 38: 2-hydroxybutyrate, 39: lysine, 40: glutamine, 41: hydroxyacetone, 42: acetoacetate, 43: methylacetoacetate, 44: reduced glutathione (GSH), 45: oxidized glutathione (GSSG), 46: malonate, 47: trimethylamine. Reprinted under the Creative Commons Attribution License (CCAL) terms from [41].

SI 1.4. Rare Applications of Metabolic Ex Vivo NMR Spectroscopy

This section is dedicated to ex vivo metabolic NMR studies of tissues and biological materials that are rarely subjected to metabolic analysis by NMR spectroscopy. In addition to excised blood vessels, this includes work on bacteria, yeast, fungus, parasites, and plant cells, as well as cells recovered from body fluids such as ascites and sperm, but also milk and serum, and metabolite assignments of general interest.

SI 1.4.1. Samples of Mammalian Origin

Blood Vessel Tissue

Anwar et al. presented an optimized procedure for extraction of human vein tissue for ^{1}H-NMR-based, untargeted metabolic profiling in conjunction

with LC-MS [42]. First, the authors tested extraction with two different ratios of methanol/water and five organic solvents for polar and unpolar-compound extraction, respectively. Based on principal component analysis of NMR and MS spectra, they found that the combination of unpolar-metabolite extraction with methyl *tert*-butyl ether/methanol (3:1), followed by extraction of polar compounds with methanol/water (1:1), was the best method for extracting metabolites from human vein tissue in terms of reproducibility and number of signals detected, in both NMR and LC-MS analysis. However, it should be noted that only the MS spectra presented were assigned, but not the NMR spectra (Supplementary Material of [42]). Moreover, a detailed list of metabolites detected was given for MS only. In particular, relevant information on lipids, in particular PLs, appears to have been gained exclusively from MS. As pointed out above, tissue extract PL analysis by NMR can only be accomplished in a meaningful way by ^{31}P-NMR spectroscopy, which, however, was not used in this work. Analysis of many PL classes and subclasses can be carried out by ^{31}P-NMR, based on differences in ^{31}P chemical shift, the latter being mostly sensitive to chemical differences in or near the polar head group of PLs. Of course, measuring individual PL species is only feasible by the more complex LC-MS that permits separation of PLs prior to MS. Consequently, the choice between NMR and MS for PL analysis depends on whether detailed knowledge of the exact PL species is needed for the scientific goal to be achieved. If this is the case, PL analysis by NMR is entirely superfluous. If only PL (sub)classes need to be quantified, PLs to be analyzed by ^{31}P-NMR need to be dissolved in a solvent different from the one given in this work [42], such that the PL head groups containing the ^{31}P nucleus become sufficiently mobile and free of complexation [4,5]. The lipid ^{1}H-NMR spectrum presented by the authors only permits identification of a few lipid classes because of strong signal overlap.

Serum lipids

Chen et al. investigated the ability of lipid-soluble nitroxides to selectively suppress the peaks of lipid resonances in ^{31}P-, ^{1}H-, and ^{13}C-NMR spectra [43]. This study was performed on serum, as part of a project aimed at using these contrast agents in vivo. The (paramagnetic) nitroxides in question were fatty acid analogs, more precisely doxyl stearates. These suppressed the methyl resonance of "choline" (typically representing the choline moieties of lipids such as PtdCho and sphingomyelin in low-density lipoproteins), and the methyl and methylene peaks of lipids in the ^{1}H-NMR spectra of serum samples. As a consequence, lactate peaks, which were not readily detected because of strong overlap with lipid methylene peaks, became clearly resolved and could be evaluated quantitatively. The ^{31}P-NMR resonance of PtdCho in the ^{31}P-NMR spectrum was suppressed by 5-doxyl stearate. In the ^{13}C-NMR spectrum, the resonances of the methyl groups of "choline" and the lipids were broadened significantly by addition of 5-doxyl stearate. Thus, it was concluded that differential suppression of lipid resonances can be employed to facilitate quantitation of lactate [43].

Skin Metabolism

An unusual application of NMR spectroscopy was the first comprehensive metabolic study of skin in a minipig model [44]. The purpose

of this work was to determine the actual metabolic state of skin following skin expander placement as performed in plastic surgery. The basic biochemical parameters for various conditions of postsurgical wound healing upon expander placement and cutaneous growth were determined by ^{1}H-NMR spectroscopy following skin extraction with perchloric acid. It was found that the lactate/alanine ratio was significantly increased in skin attached to noninflated expanders vs. control skin, indicating increased anaerobic glycolysis. Furthermore, creatine/PCr ratios were decreased in skin from inflated vs. noninflated expanders, implying an improved energetic state for stretched skin.

Ascites cells

Skog et al. reported results of ^{31}P-NMR spectroscopy measurements of energy metabolism of growing ascites tumors following addition of glucose [45]. Although ascites cells were measured in suspension, this experiment was fundamentally different from the conventional cell suspension setup. This is due to the fact that during the NMR acquisition the ascites cells were suspended in their ascites fluid taken from the host together with the cells, thus reflecting in vivo growth conditions. In conventional cell suspension experiments, cells are suspended in a buffer or in artificial cell culture medium, but not in a body fluid. The authors observed that when the ascites cells were reaching the plateau phase of growth, the ATP content and the phosphorylation potential decreased. Upon addition of glucose, the phosphorylation potential immediately increased. They concluded that the reduced phosphorylation potential was due to a limited availability of glucose, although the blood glucose concentration was found to be nearly normal. An increasing diffusion distance from the host to all parts of the tumor was identified as a possible reason for this result.

Spermatozoa

Patel et al. found in a combined ^{1}H-, ^{31}P- and ^{13}C-NMR study that arginine activated glycolysis of goat epididymal spermatozoa [46]. The underlying hypothesis was that the amino acids detected in seminal plasma play an important role in spermatozoal metabolism and motility. The epididymis was separated from the testes, and the cells from the cauda region of the epididymis were collected by gentle mincing and tweezing in Dulbecco's buffer. The cells were washed, made into a pellet by centrifugation, and then resuspended in a small amount of buffer (cell motility >60%, as ascertained by optical microscopy). Spermatozoa were incubated with different concentrations of L-arginine to determine its effect on the utilization of glucose, fructose, and pyruvate. Arginine is not consumed in these reactions, but acts as an activator. Whereas ^{31}P-NMR has been used to estimate the change of pH in the presence of different concentrations of L-arginine, ^{13}C-NMR has been used to estimate substrate consumption and lactate production. Three different isotope-labelled molecules, 1-^{13}C glucose, 1-^{13}C fructose, and 3-^{13}C pyruvate have been used as substrates. It was concluded that the presence of relatively small concentrations of L-arginine not only enhanced metabolism and spermatogenesis, but also the synthesis of ATP, an energy-rich compound essential for sperm motility. This result pointed to the

potential clinical usefulness of L-arginine when human semen with subnormal motility is utilized for artificial insemination.

Milk lipids

PLs from a different body fluid were analyzed by Garcia et al. using ^{31}P-NMR spectroscopy of milk extracts [47]. Here, the objective was to identify and quantify phospholipids in milk from four different species (human, cow, camel, and mare). Milk lipids were extracted according to a modified Folch's method: chloroform/methanol was added to milk, followed by a 0.9% NaCl / 2% acetic acid solution before centrifugation. The lower organic phase containing lipids was evaporated under nitrogen and redissolved in a ternary mixture of deuterated chloroform/methanol/aqueous solution of cesium cyclohexanediamine tetraacetic acid (CsCDTA) in H_2O. The lower organic phase of this preparation was subjected to ^{31}P-NMR analysis. The authors concluded that phospholipid fingerprints of milk determined by high-resolution ^{31}P-NMR spectroscopy were of particular practical interest for the choice of optimal ingredients to produce functional foods or dietary supplements for the prevention or treatment of specific diseases, or to produce new formulae for infants or adults in the field of artificial nutrition.

SI 1.4.2. Samples of Non-Mammalian Origin and Microorganisms

Mussels

*A*ll biological material referred to in Supplementary Materials up until here was of mammalian origin. However, it should be emphasized that in vitro NMR spectroscopy has also been applied to the metabolism of non-mammalian species. Among the more recent studies, there is a ^{1}H-NMR analysis of key metabolites of the polar metabolome of the freshwater mussel, *Elliptio complanata* [48]. Mussels were taken from their natural habitat (whose water quality was recorded), and within one minute, multiple tissue regions of interest were excised and placed in dry ice until further processing. Frozen tissues were pulverized using a blender homogenizer and incubated at 4 °C overnight in Ringer's solution. After centrifugation, the supernatant was lyophilized and redissolved in 90% H_2O / 10% D_2O containing TSP for referencing. By using principal component analysis of binned spectra (bin width: 0.04 ppm), relative concentration differences in multiple metabolites across different tissues of *E. complanata* were detected. The authors considered their study an initial step toward understanding which prominent metabolites characterize freshwater mussel tissue and how metabolomic studies may inform efforts to understand freshwater mussel physiology relevant to health and disease.

In the past few years, García-Álvarez et al. have presented two review papers on metabolomic ^{1}H-NMR analysis applied to microbiological research [49]. These investigations can be subdivided in two different classes: (i) analysis of (bio)fluids infected with a microorganism of interest (focus of García-Álvarez et al.); (ii) direct analysis of the metabolites of the microorganism of interest. Method (i) is much simpler than method (ii) because centrifugation of the microorganism-containing fluid in question is the only sample preparation step needed before lyophilization and re-dissolution in an adequate solvent. García-Álvarez et al. suggested that

because in method (i) the measurements are carried out on supernatants, there is no need to quench cell growth rapidly [50]. However, for accurate quantitative analysis of supernatants, one should consider that without rapid quenching of cell growth and metabolism, the supernatant must be separated very quickly from the cells lest the cells may continue to deplete and enrich the supernatant in metabolic substrates and catabolites, respectively, while the cell suspension is being handled when no longer under standard growth conditions. This point should be of less importance if only qualitative assessment is required, e.g., if the presence of sepsis is to be confirmed by identifying the occurrence of particular characteristic compounds in blood or other tissues. Several ^{13}C-NMR studies in the context of microbiological research have also been mentioned [49].

Bacteria

The set-up of cell suspension NMR spectroscopy has to be particularly adjusted for special cases such as measurements of bacteria suspensions. Altintas et al. have studied glucose and xylose catabolism in strains of *Zymomonas mobilis* bacteria using ^{31}P-NMR spectroscopy of intact cells [51]. These bacteria are known for their efficiency in alcoholic fermentation. Their ^{31}P-NMR measurements revealed noninvasive information about the kinetics of sugar utilization by *Z. mobilis* as well as the energetics of sugar metabolism of cells grown on glucose or xylose. In particular, uptake of glucose, fructose and xylose were investigated, in addition to time-dependent variations in intracellular pools of phosphorylated sugar metabolites, the energy status of the cells and the intracellular pH. All these parameters were also studied as a function of temperature between 4 and 30 °C. The particular challenge in this experiment was the choice of an appropriate, multi-step sample preparation protocol. Step 1: Cells grown to the late exponential phase were harvested by centrifugation at 4 °C and washed with a 100 mM MES (2-(*N*-morpholino)ethanesulfonate) buffer at pH 5.80 containing 50 mM glucose or xylose, 5 mM KH_2PO_4, 4 mM $MgCl_2$, and 2 mM Na_2EDTA (still at 4 °C). Step 2: Thereafter, cells were concentrated in an MES buffer at pH 5.80 that contained all of the compounds listed above, but at a tenfold concentration. Step 3: 2.1 mL of this cell suspension was mixed with 450 μL D_2O and 60 μL of a 1 M triethylphosphate (TEP) solution serving as a chemical-shift reference. Step 4: The resulting mixture was spiked with 400 μL of a 50% (*w*/*v*) glucose (358 mM) or xylose (430 mM) solution at the start of the NMR measurement [52]. As opposed to mammalian cells, the enzymes of these bacteria are not optimized for functioning at 37 °C, such that significant metabolism takes place even at the lower temperatures used in this study. As a result, xylose metabolism was confirmed to be slower than glucose metabolism as evidenced by much lower sugar monophosphate levels in the xylose vs. glucose experiments (for results, also see [53]). Moreover, xylose-fermenting cells were significantly less energized (nucleoside triphosphate (NTP) levels below detection threshold) than those fermenting glucose.

Moreau et al. have studied mechanisms used by *Escherichia coli* cells to survive oxidative stress when starved for glucose or phosphate [54]. Here, the *E. coli* strain ENZ361 was grown in 100 mL of MOPS medium limited in inorganic phosphate (P_i) or glucose. Exponential growth ceased abruptly after <6 h of incubation (entry into stationary phase). Thereafter, incubation was

continued for various delays between 20 min and 48 h, when the cells were treated with chloramphenicol and NaN_3 to stop metabolism. For each time point, the bacterial culture was centrifuged and resuspended twice in $MOPS_0$-NaN_3 buffer to a final volume of 500 µL, and introduced into a 5 mM NMR sample tube. ^{31}P-NMR spectra obtained at 4 °C showed that P_i-starved cells were still able to consume P_i by liberating P_i from phosphorylated products, notably through the synthesis of UDP-glucose. It was concluded that this may explain how glycolysis, which requires free P_i, could proceed in culture medium deprived of phosphate.

Yeast and Other Fungi

Himmelreich et al. presented a method for rapid identification of several clinically important yeast species of the *Candida* genus by ^{1}H-NMR spectroscopy, including a statistical classification strategy (SCS) [55]. Yeast colonies were removed from culture plates and suspended in 0.5 ml phosphate-buffered saline (pH 7.2), based on D_2O to a final concentration of 10^8 to 5×10^9 colony-forming units (CFU)/mL, prior to NMR analysis at 37 °C. Major metabolites were identified by using 2D spectra, but 1D spectra were analyzed by using a staged statistical classification strategy. Based on NMR spectra from 442 isolates of five *Candida* species, the authors concluded that this method resulted in rapid, accurate identification when compared with conventional and DNA-based identification.

An innovative device for yeast NMR spectroscopy was suggested by Zakhartsev et al. [56]. They designed a miniaturized cell agitator that fits into an 8 mM NMR probe. This cell agitator, being mounted into the instrument, is located outside of the sensitive region of the NMR coil. The device consists of two glass tubes connected in a way that, when gas is blown through these, it creates influx of cell suspension into the device, whereupon the suspension returns through apertures. This flow creates a vortex in the cell suspension while there are no moving mechanical parts or gas bubbles crossing the instrument's NMR-sensitive volume during measurement. The authors demonstrated the feasibility of this system by way of performing ^{31}P-NMR spectroscopy on a high-density *S. cerevisae* suspension being agitated by the device described. In 1 h spectra, sugar phosphates, extra and intracellular P_i, two oligophosphate peaks and one polyphosphate peak were detected. The authors suggested the addition of a second supply line to this assembly, feeding fresh medium with concentrated solutions of nutrients. A rationale for and an overview of micro-reactors for yeast and bacteria NMR (predominantly ^{31}P-NMR) spectroscopy was also made available [57].

Very recently, Judge et al. suggested to use an existing device, i.e., HR-MAS equipment, to obtain high-resolution ^{1}H-NMR spectra from fungus and lymphoid leukemia cells [58]. Although our review is not focused on HR-MAS NMR, we will briefly discuss this method because it has explicitly been designed as an alternative to extract NMR spectroscopy. Cells were placed in an HR-MAS rotor together with medium containing nutrients. Subsequently, a series of spectra was acquired at 25 °C over several hours, with a temporal resolution of about 4 min due to the relatively high resolution and sensitivity of the method. The authors point to the advantage of this continuous time-course measurement, namely that it replaces multiple cell extract measurements, each extraction performed at a different time point. Indeed, if

the scientific goal of an experiment requires measurements under the very special conditions of general, continuous depletion of nutrients, as well as general, continuous accumulation of waste products in growth medium, the suggested method generates such culture conditions for the cells and permits monitoring, by dynamic NMR spectroscopy, the metabolic changes created over time under these circumstances. However, most metabolic experiments require stable culture conditions, which can best be provided by continuous perfusion of the cells with fresh medium. Alternatively, one or two components of the culture medium may have to be changed at a time, as to be able to work under well controlled conditions. Here, the suggested method would not constitute an appropriate alternative, because only the first spectra of a given series represent metabolism under operator-controlled culture conditions. [This work uses the unusual terminology "in vivo" for the HR-MAS experiments suggested, while experiments with cell suspensions (even under perfusion) are commonly considered "in vitro" (meaning "in a test tube", as opposed to "in a living organism" which is the meaning of "in vivo")].

Parasites

Tabrizi et al. presented an extremely rare application of tissue extract NMR spectroscopy, metabolomics of parasites [59]. The parasites used were *Leishmania major* and *Leishmania tropica*. *Leishmania* promastigotes (extracellular insect-stage parasites) were extracted with perchloric acid at 4 °C. The lysed cells were centrifuged and the pH of the supernatant adjusted to pH 6.8. ^{1}H-NMR spectra were acquired after addition of 20% D_2O. Multivariate statistical analyses, including the unsupervised principal component analysis and the supervised orthogonal projections to latent structures (OPLS) discriminant analysis, were applied to identify the most significant relevant metabolites between *L. tropica* and *L. major*. The authors found that a number of readily identified amino acids best discriminated between the two groups of parasites.

Plants

Finally, plants and plant cells have been subjected to NMR spectroscopy for metabolic profiling. Peč et al. studied cell suspension cultures of *Cannabis sativa [60]*. Jasmonic acid (JA) or pectin was added to the suspension as elicitor. Elicitors are physical and chemical factors that cause physiological and morphological responses in plants; these responses are considered to be defense reactions to ensure survival, persistence and competitiveness. Subsequently, cultures were harvested every 2 days and extracted with methanol/chloroform/water. Solvents were evaporated from both the organic and the aqueous extract phases, and the dried residues were redissolved in fully deuterated solvents (chloroform and methanol/water mixture, respectively) for ^{1}H-NMR spectroscopy at 25 °C. Principal component analysis and partial least square-discriminant analysis were performed along with analysis of variance (ANOVA) statistics after binning the spectra (bin width 0.04 ppm). Based on this analysis, it was possible to discriminate *S. sativa* samples by age, elicitor and cell line (green or greenish-brown). For instance, unsaturated (saturated) fatty acids dominated in young (older) cells, and for both cell lines the pectin treatment had more effect on the metabolism

of saturated fatty acids than the JA treatment. As far as water/methanol-soluble metabolites are concerned, aromatic compounds tended to predominate in older cultures. In greenish-brown cultures, alanine GABA (γ-aminobutyric acid) and sucrose were dominant metabolites, but in green cultures leucine, glutamine, glutamic acid, aspartic acid, tryptophan and tyrosine were the dominant metabolites. The content in tyrosol, an antioxidant metabolite, was generally increased after eliciting with JA.

Datura stramonium is a plant with both poisonous and medicinal properties, and with alkaloid synthetic capacity. De-differentiation of transformed root cultures of this plant has previously been shown to cause a loss of tropane alkaloid synthetic capacity. Fliniaux et al. presented a study of the nitrogen metabolism of such de-differentiated suspension cultures, based on heteronuclear multiple bond coherence (HMBC) NMR spectroscopy [61]. For this purpose, roots or de-differentiated cells were grown with ^{15}N-labelled or unlabelled $(NH_4)_2SO_4$ and KNO_3, and extracted with 70% ethanol at room temperature for 1 h. After filtration, the crude extracts were evaporated to dryness under reduced pressure and each residue was dissolved in D_2O / H_2O, as were pooled media and washings. First, 1D ^1H-decoupled ^{15}N-NMR spectra were recorded, then $^1H/^{15}N$ HMBC spectra. *N*-acetylputrescine and GABA accumulated in the de-differentiated cultures to a much larger extent than in the root cultures. Therefore, the authors suggested that the loss of alkaloid biosynthesis was compensated by the diversion of putrescine metabolism away from the tropane pathway and toward the synthesis of GABA via *N*-acetylputrescine.

NMR metabolomics was also used to investigate the toxicity of plant products to cancer cells [62]. Emodin, which is an active ingredient of Chinese medical herbs, was reported to have anticancer, hepatoprotective, anti-inflammatory, antioxidant and antimicrobial activities. HepG2, a hepatocellular carcinoma cell line, were treated with emodin and extracted using a methanol-chloroform-water mixture. Although a large number of water-soluble compounds were detected, data from the organic phase of the extract were not reported (Figure S5). Multivariate statistical analyses such as principal-component analysis, PLS-DA and orthogonal partial least squares-discriminant analysis (OPLS-DA) resulted in differences in 33 [62] metabolites from the cytosol (culture medium) due to emodin treatment (Figure S5). Approximately eight pathways associated with these metabolites were disrupted in the emodin groups. Since emodin was also found to induce apoptosis in HepG2 cells after 12 h and 24 h of exposure, the metabolic reactions of the cells may be linked to this form of cell death.

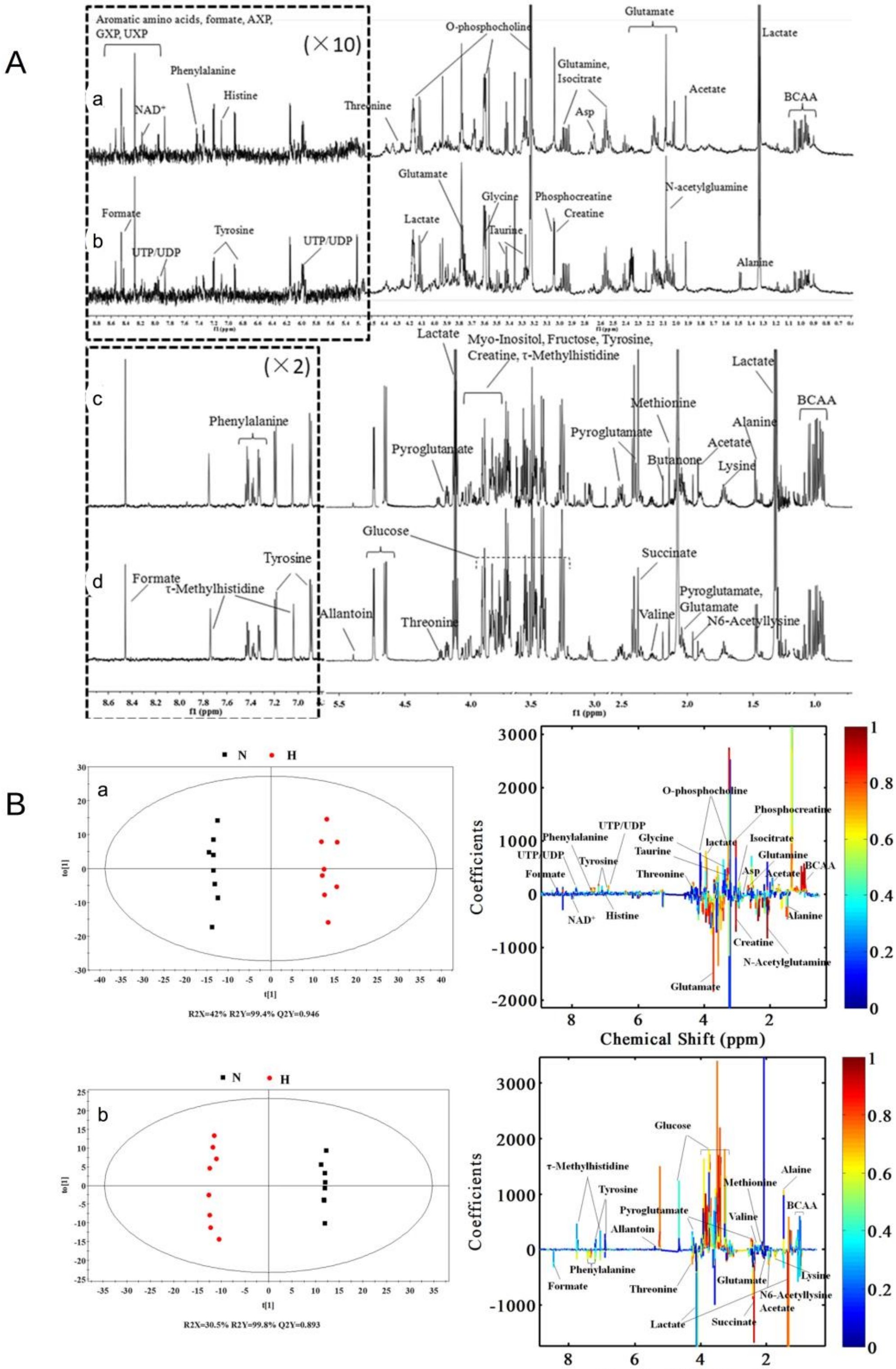

Figure S5. Panel A. Representative one-dimensional 600 MHz ^{1}H-NMR spectra (14.1 T) of HepG2 cell extracts and culture media from the normal and high-emodin groups

(see text). (**a**) spectra of cell extracts from the high-emodin group; (**b**) spectra of cell extracts from the normal group; (**c**) Spectra of cell culture media from the high-emodin group; (**d**) spectra of cell culture media from the normal group. BCAA (branched-chain amino acid); AXP (AMP, ADP, ATP); GXP (GMP, GDP; GTP); UXP (UMP, UDP, UTP). Panel B. OPLS-DA scores plots (**left**) and corresponding coefficient loading plots (**right**) derived from the ^{1}H-NMR spectra of cell extracts (**a**) and cell culture media (**b**) samples obtained from different groups. N: normal group; H: high-emodin group (100 μM). Reproduced from [62] (no permission required).

Corals

Andersson et al. published an evaluation of sample preparation methods for the analysis of reef-building corals using ^{1}H-NMR-based metabolomics [63]. The authors evaluated three important steps during sample processing of stony corals: (i) metabolite extraction, (ii) metabolism preservation, and (iii) subsampling. Previously existing coral processing methods lacked the ability to separate the coral tissues from the coral skeleton without compromising the metabolic state of the tissues. This complex biological matrix presented unique challenges when processing and extracting stony coral samples for metabolomics analysis, as extraction procedures are complicated by the large amounts of non-extractable $CaCO_3$. Of particular concern was the fact that the presence of $CaCO_3$ particles increased the difficulty of extraction tube transfer steps and, therefore, increased the likelihood of sample loss. Ultimately, the authors chose the Bligh and Dyer method because this technique makes use of two solvents only, thus minimizing the number of tube transfer steps. This is of particular importance in view of their decision to sample not only nubbins of corals, but entire coral samples (tissue plus underlying skeleton) that are then homogenized and extracted (tissue powder method). This decision was made based on the fact that the use of nubbins presents standardization and normalization challenges, as it can be difficult to accurately estimate the amount of extractable tissue present in a given nubbin. This was evidenced by the finding that tissue powder samples formed tighter clusters at principal component analysis than the nubbin samples. In addition, the authors compared freezing with or without drying as preservation methods, and chose freeze-drying because differences in metabolite profiles were minimal between the two methods.

Several of the papers discussed in this overview also contained 2D NMR spectra for assignment of signals whose molecular origin was uncertain, even though we did not describe these explicitly in all cases. Moreover, several published papers have been focusing on signal assignment relevant to tissue metabolism, although some of this work has not been performed on tissues but on biofluids. Since peak assignment is not a major focus of our review, we will not discuss this topic in detail; the interested reader is invited to consult the references given here [29,64–67].

SI 2. Essential Procedures in NMR Spectroscopic Analysis of Metabolites Based on Tissue Extracts

Metabolic in vitro NMR analysis of tissue extracts typically comprises the following steps: (i) prepare tissue culture or organism; (ii) quench metabolism; (iii) harvest tissue; (iv) destroy tissue structures and denature proteins; (v) extract solutes; (vi) separate metabolite solution from tissue debris; (vii) process metabolite solution; (viii) prepare sample for NMR analysis; (ix) set

up and run NMR data acquisition; (x) process and post-process NMR data; (xi) quantify metabolites. Note that some of these procedures may be executed simultaneously rather than sequentially; for instance, steps (ii) and (iii) combined, or steps (iii) through (v) combined. In some cases, the order of certain steps may be changed, for instance, by swapping steps (ii) and (iii); see Table S1.

Table S1. Overview of experimental procedures for metabolite analysis by NMR spectroscopy of tissue extracts. For step numbers (i) to (xi), see Section SI 2; the temporal sequence of procedural phases is ordered by letters (a to k).

	Step	Procedure	Materials	Good-Practice Hints	Refs.
**A) Soft tissue**					
**a)**	(i)	Anesthetize animal (if applicable)	Same anesthetics as for in vivo experiments can be used, such as isofluorane, ketamine/acepromazine, and many others.	Use of anesthetic may have effects on metabolic profiles; this needs to be considered when interpreting results.	[68,69]
**b)**	(ii)	Rapidly cool tissue (if applicable)	If animal tissue is to be cooled **before** excision, techniques such as funnel freezing with, or progressive insertion into liquid N_2 (dewar) should be applied where appropriate. Not usually applicable to human biopsies, except for the rarely used cryobiopsy with dedicated equipment.	External cooling/freezing only adequate for easily accessible body regions such as animal head (for brain freezing), extremities.	[39,70]
**c)**	(iii)	Rapidly excise tissue	Dedicated scissors, scalpel	If tissue metabolism was not quenched before this step, excision should be performed very quickly (within seconds rather than minutes) and immediately followed by phase **(d)** below.	[71]
**d)**	(ii)	Rapidly cool tissue	Preferably place in ice-cold organic solvent in homogenization tube (see phase **(e)** below) after quick weighing, or in liquid N_2.	For tissue to be cooled only **after** excision, cool sample immediately after phase **(c)** above. If to be placed directly in organic solvent (e.g., methanol), quickly weigh sample before this step, and immediately proceed to phase **(e)** after this step.	[39,70]

**e)**	(iv), (v)	Grind tissue	If sample was placed in organic solvent after excision, preferably use an electric tissue homogenizer. If sample was frozen in liquid N_2 after excision, add sample to a mortar partially filled with some liquid N_2 and extracting agent (e.g., PCA solution, which immediately freezes under liquid N_2), and use N_2-cooled pestle to grind tissue under liquid N_2. Extraction and homogenization in organic solvent can also be applied after pre-grinding frozen tissue in mortar under liquid N_2.	Using an electric homogenizer is faster (≤1 min) than grinding by hand (several minutes). Methanol is less efficient in precipitating proteins than PCA. Samples to be frozen in liquid N_2, can be quickly weighed either (1) before freezing, (2) frozen before addition to mortar or homogenization tube (to be preferred), or (3) after grinding (if only part of ground material is to be used for extraction). Classical glass tissue grinders are not adequate for excised tissues.	[39,72]
**f)**	(v)	Extract metabolites	First organic extraction solvent (e.g., ice-coldmethanol), or PCA solution. Samples are vortexed and left standing on ice to allow completion of extraction process. If extraction of both water-soluble metabolites and lipids is desired, methanol addition and vortexing is followed by sequential addition of cold chloroform and cold water (usually in 1:1:1 vol.), each followed by vortexing.	When tissue is homogenized in organic extraction solvent (e.g., methanol), extraction starts automatically and concomitantly with homogenization. When tissue is ground in liquid N_2 in the presence of an extraction agent (e.g., with frozen PCA solution), extraction starts after thawing of the ground slurry.	[4,14,20, 35,39]

**g)**	(v), (vi)	Centrifuge sample/solvent mixture (slurry)	High-speed centrifuge (at least 12,000 RPM, corresponding to about 12,000 RCF for a rotor radius of 7 cm). For chloroform-containing mixtures, special centrifuge tubes made of glass resisting strong centrifugal forces have to be used. For PCA extraction mixtures, use KOH to neutralize the acid in the metabolite solution, then centrifuge again.	In the case of PCA extraction, centrifugation serves to separate the acidic solution of water-soluble metabolites from denatured, precipitated proteins and cell debris (first centrifugation), along with precipitated perchlorate salt (second centrifugation). In the case of methanol/chloroform/water extraction, centrifugation serves to separate the lower, organic phase (chloroform/methanol), containing dissolved lipids, from the upper, aqueous phase (water/methanol), containing water-soluble metabolites. Here, precipitated denatured proteins and cell debris are to be found in the interphase separating the organic and aqueous phases upon centrifugation.	[39]

**h)**	(vi), (vii)	Pipet and dry metabolite solution	Solvents to be removed by<br>1) lyophilization (freeze-drying), for metabolite solutions from PCA extracts, and for aqueous phases of methanol/chloroform/water extracts upon removal of methanol (see (2) below). Before lyophilization of aqueous solutions, in the absence of methanol, divalent cations may be removed by cation exchange gel filtration with dedicated resin beads, e.g., Chelex, for line narrowing in particular in ^{31}P-NMR.<br>2) evaporation of solvent with the help of air or nitrogen bubbling through or blowing over cold lipid solutions, for organic and aqueous phases of methanol/chloroform/water extracts.	In the case of methanol/chloroform/water extraction, the aqueous phase should be lyophilized only after careful evaporation of methanol. Any non-negligible amount of methanol remaining in the aqueous phase would result in lowering the freezing point of the solution, which would prevent the solution from freezing and thus hamper proper lyophilization.<br>The removal of divalent cations by cation exchange gel filtration can be replaced with redissolving the dried sample with a cation-masking chelate (see (i) below).	[39]

**i)**	(viii)	Redissolve dried solute in solvent for NMR analysis	Solvents to be used for NMR sample preparation in analysis of:<br>1) water-soluble metabolites:<br>a) for ^{1}H-NMR: D_2O buffered with, e.g., phosphate buffer, or other ^{1}H-free pH buffer<br>b) for ^{31}P-NMR: H_2O or D_2O buffered with e.g., HEPES buffer, or other ^{31}P-free pH buffer<br>c) for other nuclei: aqueous solution buffered with any buffer not containing nuclei to be used in NMR analysis.<br>For (a) to (c), in particular for (b), divalent ions can be masked by adding at least 20 mM of an appropriate chelating agent (EDTA or CDTA with Cs^{+} (from CsCl) as counterion) to the solvent for line narrowing (see also (h) above).<br>2) phospholipids by ^{31}P-NMR: a water/chloroform/methanol-based mixture seems to be most appropriate. A homogeneous solution (one single phase) is achieved with the vol. ratio 40:10:50, with the aqueous component being slightly acidic and containing at least 20 mM of an appropriate chelating agent (EDTA or CDTA with Cs^{+} as counterion).<br>3) other lipids by ^{1}H-NMR: $CDCl_3$.	Final pH should be adjusted to about 7 or slightly acidic values for the aqueous metabolite solution and for the aqueous component of the water/chloroform/methanol-based mixture for phospholipid analysis.<br>While vol. ratios slightly different from 10:50:40 for the latter may yield excellent NMR spectral resolution, they result in the formation of two phases in the NMR sample, only one of which contains the phospholipids to be analyzed. This complicates quantitation. For generating a lock signal during ^{31}P-NMR acquisition, use $CDCl_3$ instead of $CHCl_3$.<br>Some phospholipids can be quantitated based on ^{1}H-NMR of $CDCl_3$ solutions, but analysis is rather limited due to strong peak overlap between different lipid resonances.	[6,21,39, 73–75]

**j)**	(viii), (ix)	Transfer sample to magnet and start NMR analysis	Once samples are redissolved in solvent for NMR analysis, they should be handled at 0 °C (on ice) and quickly measured at 0-4 °C to avoid metabolite degradation. Ideally, acquisition parameters should be set up with a model sample not needed for analysis, and all parameters (shim values, tune and match, temperature stability, sequence parameters including spectral width and a long acquisition time for best resolution, decoupling power, temperature stability and others) should be optimized on this test sample. ^{31}P-NMR may require thousands of transient accumulations and several hours of acquisition time to obtain good signal-to-noise ratios for low-abundance metabolites. During that time, the stability of the variable-temperature unit (VTU) must be guaranteed. External or internal reference compounds should be used for absolute metabolite quantitation.	Note that for phosphorylated compounds, absolute and relative positions (chemical shifts) of ^{31}P-NMR resonances are sensitive to extract concentration (notably for phospholipids), pH value in the aqueous component of the solvent, and measurement temperature. As always in high-resolution NMR spectroscopy, careful (re-)shimming of each new sample is crucial for best spectral resolution. External reference compounds contained in a coaxial capillary insert in the NMR tube have the advantage of being exchangeable, as a function of the nucleus to be observed. This is of interest if multiple nuclei are to be analyzed for the same sample; in this case, reference resonances not of interest do not interfere with metabolite spectra.	[4–6]

**k)**	(x), (xi)	Process, post-process and quantify NMR spectra	Careful choice of filtering parameters is of utmost importance for optimal spectral resolution. Processing software permitting resolution enhancement features such as Lorentzian–Gaussian line shape transformation should be employed.<br>Quantitation and subsequent statistical evaluation of metabolite concentrations can be performed either manually or using (semi-)automatic programs (the latter are often parts of integrated metabolomics packages).	Note that different regions of the same spectrum may require different filtering parameters, as a function of line widths. Therefore, multiple processing with different filter values should be used for quantitating different spectral regions. For each processing, the reference signal provides a new valid value for the area under the signal. While (semi-)automatic quantitation programs have the advantage of saving the operator much tedious work and time, their main disadvantage is that the operator is largely excluded from any meaningful quality control. For instance, a very weak point of current automated software is poor, imprecise quantitation of strongly overlapping peaks, in particular for small peaks overlapping with strong peaks. This results in large errors in areas under peaks and inaccurate metabolite concentrations.	[4–6,74]
**B) Cultured cells**					
**1**	*adherent cells*				
**a)**	(i) [(ii)]	Rinse cells	Rinsing liquid: preferably 4 °C buffer. If ^{31}P-NMR intended: use phosphate-free buffer. If ^{1}H-NMR intended: use solution without compounds interfering with metabolite resonances (e.g., phosphate-buffered saline, carbonate-bicarbonate buffer, or lactate-free Ringer's solution).	For maximum rinsing speed, pour (rather than pipet) rinsing liquid. Keep cells on ice during rinsing, whenever possible.	[2,14,28]

**b)**	(ii)	Quench metabolism	Add liquid N_2.	Pour N_2 into flask and spread across surface by tilting flask. Put on ice.	[12,13]
**c)**	[(iv)], (v)	Add extraction liquid and mix	For PCA extract: 0.9 N perchloric acid in H_2O. For organic extract: methanol as first solvent.	Spread PCA solution across surface by tilting flask. Put flask on ice and let melt. During melting, scrape entire flask surface with rubber policeman (cell scraper) and mix slurry thoroughly.	[2,14,21]
**d)**	(vi)	Centrifuge slurry	For PCA extracts only: use centrifuge tube at about 12,000 RPM (see A)g) above). Discard precipitate (cell debris, proteins), or use for protein quantitation. Neutralize supernatant with KOH.	After pH neutralization, re-centrifuge and discard precipitated $KClO_4$. See A)f) and A)g) above.	[12–14]
**d')**	(v), (vi)	Add further extraction liquids and mix	For organic extracts only: vortex slurry and let stand on ice for 10 min; add chloroform, vortex slurry and let stand on ice for 10 min; add water, vortex slurry and let stand on ice for 10 min. Centrifuge in glass tube at about 12,000 RPM.	Separately remove upper and lower liquid phases, avoid re-mixin. Discard precipitate of the interface (cell debris, proteins), or use for protein quantitation. See A)f) and A)g) above!	[2]
**e)**	(vii) to (xi)	As described above for tissue extracts; see A)h) to A)k)			
**2**	*single-cell suspensions*				
**a)**	(i), (iii)	Harvest /start quenching metabolism in intact cells	Centrifugation speed should not exceed a few 100 RCF (e.g., 160 RCF, corresponding to about 1,200 RPM for a rotor radius of 10 cm).	Limit RCF to avoid cell lysis. Centrifuge should be at about 4 °C to cool down medium.	[23,27,28]
**b)**	(ii), (iii)	Rinse intact cells and quench metabolism	Remove medium, re-suspend in 4 °C buffer and spin down cells twice for 8–10 min.	Centrifugation conditions as described above in B)2a).	[23,27,28]
**c)**	(iv) to (xi)	As described above for cell extracts; see B)1c) to B)1e)	Use 4 °C PCA or methanol and vortex thoroughly at 4 °C.		[23,27,28]

SI 3. References

1. Mili, M.; Panthu, B.; Madec, A.-M.; Berger, M.-A.; Rautureau, G.J.P.; Elena-Herrmann, B. Fast and ergonomic extraction of adherent mammalian cells for NMR-based metabolomics studies. *Anal. Bioanal. Chem.* **2020**, *412*, 5453–5463. https://doi.org/10.1007/s00216-020-02764-9.

2. Lutz, N.W.; Banerjee, P.; Wilson, B.J.; Ma, J.; Cozzone, P.J.; Frank, M.H. Expression of Cell-Surface Marker ABCB5 Causes Characteristic Modifications of Glucose, Amino Acid and Phospholipid Metabolism in the G3361 Melanoma-Initiating Cell Line. *PLoS ONE* **2016**, *11*, e0161803. https://doi.org/10.1371/journal.pone.0161803.
3. Mori, N.; Wildes, F.; Takagi, T.; Glunde, K.; Bhujwalla, Z.M. The Tumor Microenvironment Modulates Choline and Lipid Metabolism. *Front. Oncol.* **2016**, *6*, 262. https://doi.org/10.3389/fonc.2016.00262.
4. Lutz, N.W.; Cozzone, P.J. Multiparametric optimization of ^{31}P-NMR spectroscopic analysis of phospholipids in crude tissue extracts. 1. Chemical shift and signal separation. *Anal. Chem.* **2010**, *82*, 5433–5440.
5. Lutz, N.W.; Cozzone, P.J. Multiparametric optimization of ^{31}P-NMR spectroscopic analysis of phospholipids in crude tissue extracts. 2. Line width and spectral resolution. *Anal. Chem.* **2010**, *82*, 5441–5446.
6. Lutz, N.W.; Cozzone, P.J. Phospholipidomics by phosphorus nuclear magnetic resonance spectroscopy of tissue extracts. In *Methodologies for Metabolomics*; Lutz, N.W., Sweedler, J.V., Wevers, R.A., Eds.; Cambridge University Press: New York, NY, USA, 2013.
7. Gadiya, M.; Mori, N.; Cao, M.D.; Mironchik, Y.; Kakkad, S.; Gribbestad, I.S.; Glunde, K.; Krishnamachary, B.; Bhujwalla, Z.M. Phospholipase D1 and choline kinase-α are interactive targets in breast cancer. *Cancer Biol. Ther.* **2014**, *15*, 593–601. https://doi.org/10.4161/cbt.28165.
8. Lane, A.N.; Tan, J.; Wang, Y.; Yan, J.; Higashi, R.M.; Fan, T.W.-M. Probing the metabolic phenotype of breast cancer cells by multiple tracer stable isotope resolved metabolomics. *Metab. Eng.* **2017**, *43*, 125–136. https://doi.org/10.1016/j.ymben.2017.01.010.
9. Lin, P.; Dai, L.; Crooks, D.; Neckers, L.; Higashi, R.; Fan, T.; Lane, A. NMR Methods for Determining Lipid Turnover via Stable Isotope Resolved Metabolomics. *Metabolites* **2021**, *11*, 202. https://doi.org/10.3390/metabo11040202.
10. Lane, A.N.; Fan, T.W.-M.; Xie, Z.; Moseley, H.N.B.; Higashi, R.M. Isotopomer analysis of lipid biosynthesis by high resolution mass spectrometry and NMR. *Anal. Chim. Acta* **2009**, *651*, 201–208. https://doi.org/10.1016/j.aca.2009.08.032.
11. Tyagi, R.K.; Azrad, A.; Degani, H.; Salomon, Y. Simultaneous extraction of cellular lipids and water-soluble metabolites: Evaluation by NMR spectroscopy. *Magn. Reason. Med.* **1996**, *35*, 194–200. https://doi.org/10.1002/mrm.1910350210.
12. Lutz, N.W.; Yahi, N.; Fantini, J.; Cozzone, P.J. A new method for the determination of specific ^{13}C enrichment in phosphorylated [1-^{13}C]glucose metabolites. ^{13}C-coupled, ^{1}H-decoupled ^{31}P-NMR spectroscopy of tissue perchloric acid extracts. *Eur. J. Biochem.* **1996**, *238*, 470–475.
13. Lutz, N.W.; Yahi, N.; Fantini, J.; Cozzone, P.J. Perturbations of glucose metabolism associated with HIV infection in human intestinal epithelial cells: A multinuclear magnetic resonance spectroscopy study. *Aids* **1997**, *11*, 147–155.
14. Lutz, N.W.; Yahi, N.; Fantini, J.; Cozzone, P.J. Analysis of individual purine and pyrimidine nucleoside di- and triphosphates and other cellular metabolites in PCA extracts by using multinuclear high resolution NMR spectroscopy. *Magn. Reason. Med.* **1996**, *36*, 788–795.
15. Shen, C.Y.; Kuo, C.H.; Wang, S.Y.; Yang, W.Q.; Kuo, C.T.; Tseng, Y.J.; Tsai, M.H. Distinct metabolic changes in human lung cancer cells with differential radiation sensitivities. *Transl. Cancer Res.* **2016**, *5*, 738–747.
16. Tome, M.E.; Briehl, M.M.; Lutz, N.W. Resistance to glucocorticoid-induced apoptosis is linked to altered glucose metabolism in mouse thymoma cells. *Magn. Reson. Med.* **2002**, *15* (Suppl. 1), 87.
17. Tome, M.E.; Briehl, M.M.; Lutz, N.W. Increasing the antioxidant defense in WEHI7.2 cells results in a more tumor-like metabolic profile. *Int. J. Mol. Med.* **2005**, *15*, 497–501.
18. Tome, M.E.; Lutz, N.W.; Briehl, M.M. Overexpression of catalase or Bcl-2 delays or prevents alterations in phospholipid metabolism during glucocorticoid-induced apoptosis in WEHI7.2 cells. *Biochim. Biophys. Acta* **2003**, *1642*, 149–162.
19. Tome, M.E.; Lutz, N.W.; Briehl, M.M. Overexpression of catalase or Bcl-2 alters glucose and energy metabolism concomitant with dexamethasone resistance. *Biochim. Biophys. Acta* **2004**, *1693*, 57–72. https://doi.org/10.1016/j.bbamcr.2004.05.004.
20. Lutz, N.W.; Tome, M.E.; Aiken, N.R.; Briehl, M.M. Changes in phosphate metabolism in thymoma cells suggest mechanisms for resistance to dexamethasone-induced apoptosis. A ^{31}P-NMR spectroscopic study of cell extracts. *NMR Biomed.* **2002**, *15*, 356–366.
21. Lutz, N.W.; Tome, M.E.; Cozzone, P.J. Early changes in glucose and phospholipid metabolism following apoptosis induction by IFN-gamma/TNF-alpha in HT-29 cells. *FEBS Lett.* **2003**, *544*, 123–128.
22. Guo, J.; Higashi, K.; Yokota, H.; Nagao, Y.; Ueda, Y.; Kodama, Y.; Oguchi, M.; Taki, S.; Tonami, H.; Yamamoto, I. In vitro proton magnetic resonance spectroscopic lactate and choline measurements. *J. Nucl. Med.* **2004**, *45*, 1334–1339.

23. Viola, A.; Lutz, N.W.; Maroc, C.; Chabannon, C.; Julliard, M.; Cozzone, P.J. Metabolic effects of photodynamically induced apoptosis in an erythroleukemic cell line. A ^{31}P-NMR spectroscopic study of Victo-ria-Blue-BO-sensitized TF-1 cells. *Int. J. Cancer* **2000**, *85*, 733–739.
24. Lutz, N.W. From metabolic to metabolomic NMR spectroscopy of apoptotic cells. *Metabolomics* **2005**, *1*, 251–268.
25. Lutz, N.W. Contributions of metabol(om)ic NMR spectroscopy to the investigation of apoptosis. *C. R. Chim.* **2006**, *9*, 445–451. https://doi.org/10.1016/j.crci.2005.06.017.
26. Shedd, S.F.; Lutz, N.W.; Hull, W.E. The influence of medium formulation on phosphomonoester and UDP-hexose levels in cultured human colon tumor cells as observed by ^{31}P-NMR spectroscopy. *NMR Biomed.* **1993**, *6*, 254–263.
27. Franks SE, Kuesel AC, Lutz NW, Hull WE. 31P MRS of human tumor cells: effects of culture media and conditions on phospholipid metabolite concentrations. Anticancer Res 1996;16:1365–74.
28. Lutz, N.W.; Franks, S.E.; Frank, M.H.; Pomer, S.; Hull, W.E. Investigation of multidrug resistance in cultured human renal cell carcinoma cells by ^{31}P-NMR spectroscopy and treatment survival assays. *Magn. Reson. Mater. Phys. Biol. Med.* **2005**, *18*, 144–161.
29. Lutz, N.W.; Hull, W.E. Assignment and pH dependence of the ^{19}F-NMR resonances from the fluorouracil anabolites involved in fluoropyrimidine chemotherapy. *NMR Biomed.* **1999**, *12*, 237–248.
30. Lutz, N.W.; Naser-Hijazi, B.; Koroma, S.; Berger, M.R.; Hull, W.E. Fluoropyrimidine chemotherapy in a rat model: Comparison of fluorouracil metabolite profiles determined by high-field ^{19}F-NMR spectroscopy of tissues ex vivo with therapy response and toxicity for locoregional vs systemic infusion protocols. *NMR Biomed.* **2004**, *17*, 101–131. https://doi.org/10.1002/nbm.880.
31. Loewenbrück, K.F.; Fuchs, B.; Hermann, A.; Brandt, M.; Werner, A.; Kirsch, M.; Schwarz, S.; Schwarz, J.; Schiller, J.; Storch, A. Proton MR spectroscopy of neural stem cells: Does the proton-NMR peak at 1.28 ppm function as a biomarker for cell type or state? *Rejuvenation Res.* **2011**, *14*, 371–381. https://doi.org/10.1089/rej.2010.1102.
32. Manganas, L.N.; Zhang, X.; Li, Y.; Hazel, R.D.; Smith, S.D.; Wagshul, M.E.; Henn, F.; Benveniste, H.; Djurić, P.M.; Enikolopov, G.; et al. Magnetic resonance spectroscopy identifies neural progenitor cells in the live human brain. *Science* **2007**, *318*, 980–985. https://doi.org/10.1126/science.1147851.
33. Sander, P.R.; Hau, P.; Koch, S.; Schütze, K.; Bogdahn, U.; Kalbitzer, H.R.; Aigner, L. Stem cell metabolic and spectroscopic profiling. *Trends Biotechnol.* **2013**, *31*, 204–213. https://doi.org/10.1016/j.tibtech.2013.01.008.
34. Kuesel, A.C.; Graschew, G.; Hull, W.E.; Lorenz, W.; Thielmann, H.W. ^{31}P-NMR studies of cultured human tumor cells. Influence of pH on phospholipid metabolite levels and the detection of cytidine 5′-diphosphate choline. NMR Biomed. **1990**, *3*, 78–89. https://doi:10.1002/nbm.1940030206.
35. Lutz, N.W.; Fernandez, C.; Pellissier, J.F.; Cozzone, P.J.; Beraud, E. Cerebral biochemical pathways in experimental autoimmune encephalomyelitis and adjuvant arthritis: A comparative metabolomic study. *PLoS ONE* **2013**, *8*, e56101.
36. Viola, A.; Saywell, V.; Villard, L.; Cozzone, P.J.; Lutz, N.W. Metabolic fingerprints of altered brain growth, osmoregulation and neurotransmission in a Rett syndrome model. *PLoS ONE* **2007**, *2*, e157.
37. Saywell, V.; Viola, A.; Confort-Gouny, S.; Le Fur, Y.; Villard, L.; Cozzone, P.J. Brain magnetic resonance study of Mecp2 deletion effects on anatomy and metabolism. *Biochem. Biophys. Res. Commun.* **2006**, *340*, 776–783.
38. Kostidis, S.; Addie, R.D.; Morreau, H.; Mayboroda, O.A.; Giera, M. Quantitative NMR analysis of intra- and extracellular metabolism of mammalian cells: A tutorial. *Anal. Chim. Acta* **2017**, *980*, 1–24 https://doi:10.1016/j.aca.2017.05.011.
39. Lutz, N.W.; Béraud, E.; Cozzone, P.J. Metabolomic analysis of rat brain by high resolution nuclear magnetic resonance spectroscopy of tissue extracts. *J. Vis. Exp.* **2014**, *91*, e51829.
40. Álvarez-Guerra, E.D.; Parkes, H.G.; Bell, J.D. Estudio comparado de dos métodos de desproteinización para la evaluación de sangre total y plasma mediante espectroscopia de resonancia magnética. *Bioquimia* **2006**, *31*, 59–68.
41. Gómez-Archila, L.G.; Palomino-Schätzlein, M.; Zapata-Builes, W.; Galeano, E. Development of an optimized method for processing peripheral blood mononuclear cells for ^{1}H-nuclear magnetic resonance-based metabolomic profiling. *PLoS ONE* **2021**, *16*, e0247668. https://doi.org/10.1371/journal.pone.0247668.
42. Anwar, M.A.; Vorkas, P.A.; Li, J.V.; Shalhoub, J.; Want, E.J.; Davies, A.H.; Holmes, E. Optimization of metabolite extraction of human vein tissue for ultra performance liquid chromatography-mass spectrometry and nuclear magnetic resonance-based un-targeted metabolic profiling. *Analyst* **2015**, *140*, 7586–7597. https://doi.org/10.1039/c5an01041a.
43. Chen K, Lutz NW, Wehrle JP, Glickson JD, Swartz HM. Selective suppression of lipid resonances by lipid-soluble nitroxides in NMR spectroscopy. Magn Reson Med 1992;25:120–127 doi: 10.1002/mrm.1910250112.
44. Lutz, N.W.; Confort-Gouny, S.; Casanova, D.; Andrac-Meyer, L.; Magalon, G.; Cozzone, P.J. Conditions of wound healing and cutaneous growth affect metabolic performance of skin following plastic surgery. *Wound Repair Regen.* **2007**, *15*, 491–496.

45. Skog, S.; Ericsson, A.; Nordell, B.; Nishida, T.; Tribukait, B. ^{31}P-NMR-spectroscopy measurements of energy metabolism of in vivo growing ascites tumours following addition of glucose. *Acta Oncol.* **1989**, *28*, 277–281. https://doi.org/10.3109/02841868909111263.
46. Patel, A.B.; Srivastava, S.; Phadke, R.S.; Govil, G. Arginine activates glycolysis of goat epididymal spermatozoa: An NMR study. *Biophys. J.* **1998**, *75*, 1522–1528. https://doi.org/10.1016/S0006-3495(98)74071-8.
47. Garcia, C.; Lutz, N.W.; Confort-Gouny, S.; Cozzone, P.J.; Armand, M.; Bernard, M. Phospholipid fingerprints of milk from different mammalians determined by ^{31}P-NMR: towards specific interest in human health. *Food Chem.* **2012**, *135*, 1777–1783. https://doi.org/10.1016/j.foodchem.2012.05.111.
48. Hurley-Sanders, J.L.; Levine, J.F.; Nelson, S.A.C.; Law, J.M.; Showers, W.J.; Stoskopf, M.K. Key metabolites in tissue extracts of Elliptio complanata identified using ^{1}H nuclear magnetic resonance spectroscopy. *Conserv. Physiol.* **2015**, *3*, cov023. https://doi.org/10.1093/conphys/cov023.
49. García-Álvarez, L.; Busto, J.H.; Peregrina, J.M.; Fernández Recio, M.A.; Avenoza, A.; Oteo, J.A. Nuclear magnetic resonance applied to antimicrobial drug susceptibility. *Future Microbiol.* **2013**, *8*, 537–547. https://doi.org/10.2217/fmb.13.8.
50. García-Álvarez, L.; Busto, J.H.; Peregrina, J.M.; Avenoza, A.; Oteo, J.A. Applications of ^{1}H Nuclear Magnetic Resonance Spectroscopy in Clinical Microbiology. In *Applications of Molecular Spectroscopy to Current Research in the Chemical and Biological Sciences*; Stauffer, M.T., Ed.; IntechOpen: Rijeka, Croatia, 2016; pp. 281–294.
51. Altintas, M.M.; Eddy, C.; Davis, M.; Zhang, M.; McMillan, J.D.; Ailion, D.S. ^{31}P-Nuclear Magnetic Resonance Studies of Sugar Metabolism in *Zymomonas mobilis*. Available online: www.nrel.gov (accessed on June 25, 2022).
52. Strohhäcker, J.; de Graaf, A.A.; Schoberth, S.M.; Wittig, R.M.; Sahm, H. ^{31}P-Nuclear magnetic resonance studies of ethanol inhibition in *Zymomonas mobilis*. *Arch. Microbiol.* **1993**, *159*, 484–490. https://doi.org/10.1007/BF00288598.
53. Kim, I.S.; Barrow, K.D.; Rogers, P.L. Kinetic and nuclear magnetic resonance studies of xylose metabolism by recombinant *Zymomonas mobilis* ZM4(pZB5). *Appl. Environ. Microbiol.* **2000**, *66*, 186–193. https://doi.org/10.1128/AEM.66.1.186-193.2000.
54. Moreau, P.L.; Gerard, F.; Lutz, N.W.; Cozzone, P. Non-growing *Escherichia coli* cells starved for glucose or phosphate use different mechanisms to survive oxidative stress. *Mol. Microbiol.* **2001**, *39*, 1048–1060.
55. Himmelreich, U.; Somorjai, R.L.; Dolenko, B.; Lee, O.C.; Daniel, H.-M.; Murray, R.; Mountford, C.E.; Sorrell, T.C. Rapid identification of Candida species by using nuclear magnetic resonance spectroscopy and a statistical classification strategy. *Appl. Environ. Microbiol.* **2003**, *69*, 4566–4574. doi: 10.1128/AEM.69.8.4566-4574.2003.
56. Zakhartsev, M.; Bock, C. Miniaturized device for agitating a high-density yeast suspension that is suitable for in vivo nuclear magnetic resonance applications. *Anal. Biochem.* **2010**, *397*, 244–246. https://doi.org/10.1016/j.ab.2009.10.011.
57. Shanks, J.V. In situ NMR systems. *Curr. Issues Mol. Biol.* **2001**, *3*, 15–26.
58. Judge, M.T.; Wu, Y.; Tayyari, F.; Hattori, A.; Glushka, J.; Ito, T.; Arnold, J.; Edison, A.S. Continuous in vivo Metabolism by NMR. *Front. Mol. Biosci.* **2019**, *6*, 26. https://doi.org/10.3389/fmolb.2019.00026.
59. Tabrizi, F.; Seyyed Tabaei, S.J.; Ali Ahmadi, N.; Arefi Oskouie, A. A Nuclear Magnetic Resonance-Based Metabolomic Study to Identify Metabolite Differences between Iranian Isolates of Leishmania major and Leishmania tropica. *Iran. J. Med. Sci.* **2021**, *46*, 43–51. https://doi.org/10.30476/ijms.2019.82120.0.
60. Pec, J.; Flores-Sanchez, I.J.; Choi, Y.H.; Verpoorte, R. Metabolic analysis of elicited cell suspension cultures of *Cannabis sativa* L. by ^{1}H-NMR spectroscopy. *Biotechnol. Lett.* **2010**, *32*, 935–941. https://doi.org/10.1007/s10529-010-0225-9.
61. Fliniaux, O.; Mesnard, F.; Grandic, S.R.; Baltora-Rosset, S.; Bienaimé, C.; Robins, R.J.; Fliniaux, M. Altered nitrogen metabolism associated with de-differentiated suspension cultures derived from root cultures of Datura stramonium studied by heteronu-clear multiple bond coherence (HMBC) NMR spectroscopy. *J. Exp. Bot.* **2004**, *55*, 1053–1060. https://doi.org/10.1093/jxb/erh119.
62. Chen, C.; Gao, J.; Wang, T.-S.; Guo, C.; Yan, Y.-J.; Mao, C.-Y.; Gu, L.-W.; Yang, Y.; Li, Z.-F.; Liu, A. NMR-based Metabolomic Techniques Identify the Toxicity of Emodin in HepG2 Cells. *Sci. Rep.* **2018**, *8*, 9379. https://doi.org/10.1038/s41598-018-27359-4.
63. Andersson, E.R.; Day, R.D.; Loewenstein, J.M.; Woodley, C.M.; Schock, T.B. Evaluation of Sample Preparation Methods for the Analysis of Reef-Building Corals Using ^{1}H-NMR-Based Metabolomics. *Metabolites* **2019**, *9*, 32. https://doi.org/10.3390/metabo9020032.
64. Lutz, N.W.; Maillet, S.; Nicoli, F.; Viout, P.; Cozzone, P.J. Further assignment of resonances in ^{1}H-NMR spectra of cerebrospinal fluid (CSF). *FEBS Lett.* **1998**, *425*, 345–351.
65. Lutz, N.W.; Viola, A.; Malikova, I.; Confort-Gouny, S.; Ranjeva, J.-P.; Pelletier, J.; Cozzone, P.J. A branched-chain organic acid linked to multiple sclerosis: First identification by NMR spectroscopy of CSF. *Biochem. Biophys. Res. Commun.* **2007**, *354*, 160–164.

66. Lutz, N.W.; Cozzone, P.J. Metabolic profiling in multiple sclerosis and other disorders by quantitative analysis of cerebrospinal fluid using nuclear magnetic resonance spectroscopy. *Curr. Pharm. Biotechnol*. **2011**, *12*, 1016–1025. https://doi.org/10.2174/138920111795909122.
67. Maillet, S.; Vion-Dury, J.; Confort-Gouny, S.; Nicoli, F.; Lutz, N.W.; Viout, P.; Cozzone, P.J. Experimental protocol for clinical analysis of cerebrospinal fluid by high resolution proton magnetic resonance spectroscopy. Brain Res Brain Res Protoc **1998**, *3*, 123–134. https://doi.org/10.1016/s1385-299x(98)00033-6.
68. Dinis-Oliveira, R.J. Metabolic Profiles of Propofol and Fospropofol: Clinical and Forensic Interpretative Aspects. *Biomed Res. Int.* **2018**, *2018*, 16 pages. https://doi:10.1155/2018/6852857.
69. Makaryus, R.; Lee, H.; Yu, M.; Zhang, S.; Smith, S.D.; Rebecchi, M.; Glass, P.S.; Benveniste, H. The metabolomic profile during isoflurane anesthesia differs from propofol anesthesia in the live rodent brain. *J. Cereb. Blood Flow Metab.* **2011**, *31*, 1432–1442. https://doi:10.1038/jcbfm.2011.1.
70. Haukaas, T.H.; Moestue, S.A.; Vettukattil, R.; Sitter, B.; Lamichhane, S.; Segura, R.; Giskeødegård, G.F.; Bathen, T.F. Impact of Freezing Delay Time on Tissue Samples for Metabolomic Studies. *Front. Oncol.* **2016**, *6*, 17. https://doi.org/10.3389/fonc.2016.00017.
71. Saoi, M.; Britz-McKibbin, P. New Advances in Tissue Metabolomics: A Review. *Metabolites* **2021**; *11*, 672. https://doi:10.3390/metabo11100672.
72. Zelentsova, E.A.; Yanshole, V.V.; Tsentalovich, Y.P. A novel method of sample homogenization with the use of a microtome-cryostat apparatus. *RSC Adv*. **2019**, *9*, 37809–37817 https://doi:10.1039/C9RA06808B.
73. Lin, C.Y.; Wu, H.; Tjeerdema, R.S.; Viant, M.R. Evaluation of metabolite extraction strategies from tissue samples using NMR metabolomics. *Metabolomics* **2007**, *3*, 55–67. https://doi.org/10.1007/s11306-006-0043-1.
74. Lutz, N.W.; Cozzone, P.J. Principles of multiparametric optimization for phospholipidomics by ^{31}P-NMR spectroscopy. *Biophys. Rev.* **2013**, *5*, 295–304.
75. Lutz, N.W.; Sweedler, J.V.; Wevers, R.A. *Methodologies for Metabolomics: Experimental Strategies and Techniques*, 1st ed.; Cambridge University Press: Cambridge, UK, 2013.
